# Multitasking Pharmacophores Support Cabotegravir-Based Long-Acting HIV Pre-Exposure Prophylaxis (PrEP)

**DOI:** 10.3390/molecules29020376

**Published:** 2024-01-11

**Authors:** Zheng Wan, Man Shi, Yanqing Gong, Massimo Lucci, Jinjin Li, Jiahai Zhou, Xiao-Liang Yang, Moreno Lelli, Xiao He, Jiafei Mao

**Affiliations:** 1 Shanghai Engineering Research Center of Molecular Therapeutics and New Drug Development, Shanghai Frontiers Science Center of Molecule Intelligent Syntheses, School of Chemistry and Molecular Engineering, East China Normal University, Shanghai 200062, China; 2 State Key Laboratory of New Drug and Pharmaceutical Process, Shanghai Institute of Pharmaceutical Industry, China State Institute of Pharmaceutical Industry, 285 Gebaini Road, Shanghai 201203, China; gongyanqing@sinopharm.com; 3 C.I.R.M.M.P.—Consorzio Interuniversitario Risonanze Magnetiche di Metallo Proteine, Via L. Sacconi 6, 50019 Sesto Fiorentino, Firenze, Italy; lucci@cerm.unifi.it; 4 Key Laboratory for Thin Film and Microfabrication of Ministry of Education, Department of Micro/Nano-electronics, Shanghai Jiao Tong University, Shanghai 200240, China; 5 CAS Key Laboratory of Quantitative Engineering Biology, Shenzhen Institute of Synthetic Biology, Shenzhen Institute of Advanced Technology, Chinese Academy of Sciences, Shenzhen 518055, China; jiahai@siat.ac.cn; 6 State Key Laboratory of Coordination Chemistry and Jiangsu Key Laboratory of Advanced Organic Materials, School of Chemistry and Chemical Engineering, Nanjing University, Nanjing 210023, China; 7 Department of Chemistry “Ugo Schiff”, Magnetic Resonance Center (CERM), University of Florence, Via della Lastruccia 3, 50019 Sesto Fiorentino, Florence, Italy; 8 New York University–East China Normal University Center for Computational Chemistry, New York University Shanghai, Shanghai 200062, China; 9 Beijing National Laboratory for Molecular Sciences (BNLMS), Institute of Chemistry, Chinese Academy of Sciences, Zhongguancun North First Street 2, Beijing 100190, China; 10 Center for Physicochemical Analysis and Measurement, Institute of Chemistry, Chinese Academy of Sciences, Zhongguancun North First Street 2, Beijing 100190, China

**Keywords:** HIV pre-exposure prophylaxis, cabotegravir, X-ray diffraction, NMR, DFT, AF-QM/MM

## Abstract

Cabotegravir is an integrase strand transfer inhibitor (INSTI) for HIV treatment and prevention. Cabotegravir-based long-acting pre-exposure prophylaxis (PrEP) presents an emerging paradigm for infectious disease control. In this scheme, a combination of a high efficacy and low solubility of anti-infection drugs permits the establishment of a pharmaceutical firewall in HIV-vulnerable groups over a long period. Although the structure-activity-relationship (SAR) of cabotegravir as an INSTI is known, the structural determinants of its low solubility have not been identified. In this work, we have integrated multiple experimental and computational methods, namely X-ray diffraction, solid-state NMR (SSNMR) spectroscopy, solution NMR spectroscopy, automated fragmentation (AF)-QM/MM and density functional theory (DFT) calculations, to address this question. The molecular organization of cabotegravir in crystal lattice has been determined. The combination of very-fast magic-angle-sample-spinning (VF MAS) SSNMR and solution NMR, as supported by AF-QM/MM and DFT calculations, permits the identification of structural factors that contribute to the low aqueous solubility of cabotegravir. Our study reveals the multitasking nature of pharmacophores in cabotegravir, which controls the drug solubility and, meanwhile, the biological activity. By unraveling these function-defining molecular features, our work could inspire further development of long-acting HIV PrEP drugs.

## 1. Introduction

Human immunodeficiency virus (HIV) causes a chronic and progressive infection that eventually reaches the stage of acquired immune deficiency syndrome (AIDS) [1,2,3,4]. The global pandemic of HIV infection and AIDS has been claiming a tremendous number of lives and has led to heavy socioeconomic burdens. HIV infection features robust latent virus reservoirs that are extremely difficult to eradicate, presenting a major challenge to HIV treatment. At the moment, no effective and ubiquitous cure is available for HIV infection and AIDS. Therefore, the most effective and economical strategy for containing the HIV/AIDS pandemic is focused on disease prevention. Unfortunately, the conventional approaches, such as infection tracing, blood screening, HIV-pregnancy support and safe sex, seem inadequate for containing the spread of HIV infections in many developing countries, high-risk groups and vulnerable populations. Additional approaches are therefore urgently required for limiting the ongoing HIV/AIDS pandemic. Pre-exposure prophylaxis (PrEP), an emerging paradigm in infectious disease control, is currently achieving significant success in HIV prevention [5,6,7,8,9]. HIV PrEP focuses on building pharmaceutical firewalls in human bodies via vaccination and/or prevention-purposed medication prior to HIV exposure. A key factor determining the efficacy of such approaches is the adherence, namely how frequent PrEP receivers follow the desired dosing scheme. In practice, a PrEP scheme requiring frequent drug intake could suffer from more intermittent drop-outs and potential failures. Such a scheme may also increase economic burdens as well as the risks of side effects and HIV escaping mutations. To resolve these issues, long-acting HIV PrEP drugs with low dosing frequency are highly desired.

**Figure 1:**
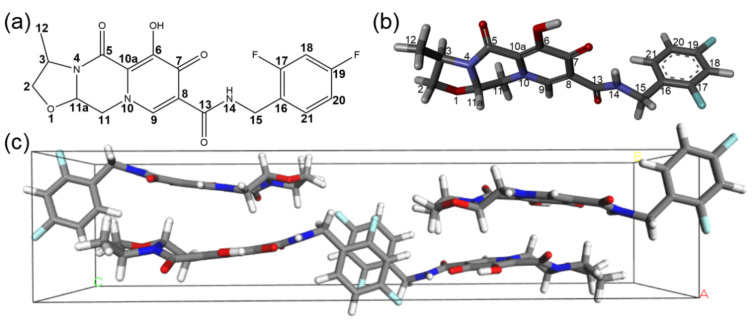
Structure of cabotegravir. (**a**) The molecular formula of cabotegravir. (**b**) The molecular structure of cabotegravir. The gray, white, green, red and blue balls represent the carbon, hydrogen, fluorine, oxygen and nitrogen atoms, respectively. (**c**) The unit cell of cabotegravir along the direction of a-axis with a space group of P2_1_2_1_2_1_ determined via XRD.

The recent development of cabotegravir (Figure 1a) is a milestone in long-acting HIV PrEP [10,11]. Cabotegravir is an integrase strand transfer inhibitor (INSTI). Its solubility is very low (about 30 μg/mL, <0.1 mM) [11], and at this solubility most drug candidates would have been rejected. Even at such low bioavailable concentrations, this drug still shows excellent HIV inhibition potency due to its high efficacy (with an IC_50_ at 0.5 nM in the pseudotyped virus assay) [12,13]. When formulated as nanocrystals, the combination of low solubility and high potency permits the establishment of a long-lasting pharmacological barrier against the genome integration of HIV virions. The PrEP efficacy of cabotegravir can be sustained for months, which leads to a new HIV prevention scheme with a much lower dropout rate and significantly higher accessibility. Recently, cabotegravir has been approved by the FDA and EPA for clinical use in HIV PrEP. The success of cabotegravir has also encouraged other new long-acting HIV PrEP schemes [14,15].

Despite the success of cabotegravir in HIV PrEP campaigns, the molecular features underlying its long-acting efficacy have not been fully resolved. A number of the structures of cabotegravir analogues bound to HIV intasome complexes have been already determined, providing grounds for understanding their extraordinary anti-HIV activities [13]. However, only one crystal structure of cabotegravir analogues (dolutegravir) is currently available (CSD Entry: KILNEA) [12]. This structure was obtained for the water-soluble sodium salt form of the drug. Therefore, it could not address directly the unique low solubility of these drugs, a key pharmaceutical feature pivotal for the long-acting HIV PrEP. To bridge this gap, we have first solved the crystal structure of cabotegravir. The molecular differences between crystal and solution samples were then mapped by combining very-fast magic-angle-sample-spinning solid-state NMR (VF MAS SSNMR) and solution NMR spectroscopy. Supported by automated fragmentation quantum mechanics/molecular mechanics (AF-QM/MM) calculations, we have further defined the key structural moieties responsible for the low solubility of cabotegravir. Our studies have revealed the functional roles of cabotegravir pharmacophores in controlling the drug’s solubility, which could inform rational drug design for next-generation long-acting HIV PrEP.

## 2. Results and Discussion

### 2.1. The Crystal Structure of Cabotegravir

To elucidate the conformation and the molecular organization of cabotegravir in crystalline solids, we have determined its crystal structure (Figure 1b,c) using single-crystal X-ray diffraction (XRD). The crystals were obtained using the standard solvent evaporation technique. Details about the XRD data collection, processing and structural refinement can be found in Table 1. The detailed data of the refined structure can be found in Tables S1–S4. As shown in Figure 1c, cabotegravir molecules are arranged with a space group of P2_1_2_1_2_1_ and a unit cell asymmetric unit number of Z = 4. Our crystal structure shows unique structural features of cabotegravir molecules and their intermolecular packing.

First, the large-sized fused ring moiety is locked in the same plane of the amide linker by an intramolecular H-bond between the carbonyl group (C10-O2) on the pyridine ring and the amine group (N1 = H_N1_) in the amide linker. The distance of this H-bond is 1.779 Å (O2 to H_N1_). This conformation permits the carbonyl group (C8 = O1) in the amide linker to form an intermolecular H-bond with hydroxyl group (O3-H_O3_) on the pyridine ring. The distance of this intermolecular H-bond is 1.729 Å (O1 to H_O3_). Via these interactions, the fused ring moieties and the amide linkers are organized in sheets, which further intercalates anti-parallelly.

Second, the piperazine ring is in a usually less stable boat conformation. The lack of axial substitution groups on four out of six piperazine ring backbone atoms, namely N2, N3, C12 and C13, makes the boat conformation possible. The boat conformation of the piperazine ring is also necessary for accommodating a five-membered oxazoline ring that is fused to the piperazine ring. The oxazoline ring is in a stable chair conformation. The axial hydrogens on the piperazine and oxazoline ring, together with the equatorial methyl group on the oxazoline ring, set the distance (3.65 Å) between the sheet of the fused rings that are stacked in an antiparallel fashion.

Third, the meta-difluorobenzene rings are rotated to almost perpendicular to the fused ring-amide link moieties. This conformation permits a partial and antiparallel stacking of these aromatic difluorobenzene rings in channels between the extended sheets formed by the fused ring moieties. The inter-place distance between the difluorobenzene rings is about 3.75 Å, which is significantly larger than the 3.5 Å threshold and indicates a rather weak packing.

Previously the crystal structure of the sodium salt form of dolutegravir, a cabotegravir analogue, was reported (CSD Entry: KILNEA) [12]. As shown in Figure S1, in this crystal structure, the fused ring moieties are organized as dimers by sodium ions via the peripheral oxygen sites (O2, O3, O4). The six oxygens from two dolutegravir chelate two shared sodium ions in a near planar conformation. Interestingly, the carbonyl oxygen (O1) of the amide linker serves as an axial ligand of one sodium ion. Since these oxygens are extensively involved in the interaction with sodium ions, they are not involved in intra- or intermolecular H-bond interactions and consequently no sheet structures are formed by the fused rings. The meta-difluorobenzene rings are tilted to about 93.6° to the fused ring moiety and do not stack as closely as those in cabotegravir crystals. The molecular organizations are drastically different between the non-salt and salt forms of this family of drugs.

The structure of the cabotegravir crystal is stabilized by only a few weak interactions, including intra- and intermolecular H-bonds, aromatic ring stacking and van der Waals forces. Moreover, it seems that these weak interactions are mutually required for making the molecular structure compatible to the intermolecular packing in crystals. Such a molecular organization is rather unique, which explains the failure of a previous effort in the ab initio prediction of the crystal structure of cabotegravir [16]. Besides the H-bond and ring stacking described above, the van der Waals forces could also contribute to the overall stability of the crystal lattice. Fluorine atoms are highly electronegative, resulting in a more tightly held electron cloud compared to other atoms. This leads to a stronger dipole-induced dipole interaction between a fluorine atom and nearby methyl methylene and difluorobenzene groups in neighboring molecules. The presence of fluorine atoms in cabotegravir therefore enhances the van der Waals forces between adjacent molecules and contributes to the overall stability of the crystal lattice. Other van der Waals interactions involving hydrogens could further improve the overall stability of the crystal lattice by contributing to the dispersion forces and inducing temporary dipoles in neighboring molecules. The intermolecular distance between H18 and the hydrogens on the methyl group of other molecules is 2.432 Å, while the distance between H18 and the H on C2 is 2.331 Å. More details for the measured lattice parameters, atomic coordinates, bond lengths, torsion angles, and hydrogen bonds are provided in Table 1 and Tables S1–S4 of the Supporting Information, respectively.

### 2.2. Optimizing the Chemical Shift Prediction for Cabotegravir

Our XRD characterization of cabotegravir has revealed some molecular features that could be relevant to the low water solubility of this drug. To better address the mechanism of the low water solubility of cabotegravir, we have aimed to map the structural differences of cabotegravir passing from solids to solution. We have chosen NMR spectroscopy for this purpose as the chemical shift, an NMR parameter that could be obtained feasibly in both solids and solution, is highly suitable for reporting the conformational changes of molecules [17,18,19,20,21,22,23]. Chemical shifts could be influenced by other factors besides the molecular structural changes, in particular, the polarity change of the molecular environments (e.g., solvent vs. crystal lattice) and the intermolecular packing. To resolve such ambiguities and to improve the NMR data interpretability, we first optimized the DFT-based chemical shift prediction directly on cabotegravir microcrystals by combining SSNMR spectroscopy and AF-QM/MM calculations.

**Figure 2:**
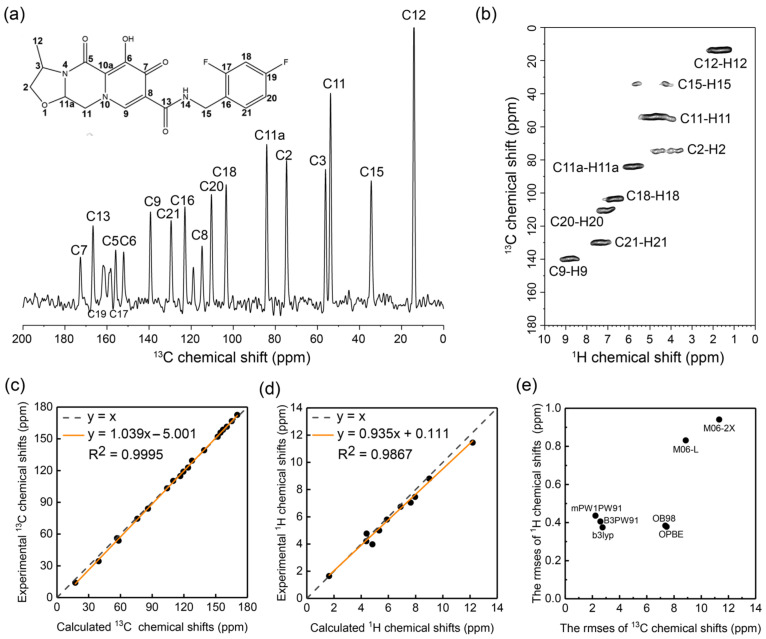
SSNMR characterization of cabotegravir microcrystalline powders and AF-QM/MM-based selection of DFT functionals for chemical shift prediction for cabotegravir. (**a**) The ^1^H-^13^C CP 1D ^13^C NMR spectrum shows well-resolved signals. The chemical shift assignments are indicated. (**b**) The CP-based ^1^H-detected ^1^H-^13^C 2D correlation spectrum shows all directly bonded C-H pairs. (**c**) The correlation of ^13^C chemical shifts between the experimental and calculated values using the AF-QM/MM method at the mPW1PW91/6-31G** level. (**d**) The correlation of ^1^H chemical shifts between the experimental and calculated values using the AF-QM/MM method at the B3LYP/6-31G** level. (**e**) The performance of different density functionals with the 6-31G** basis set in the AF-QM/MM calculation of ^13^C and ^1^H chemical shifts as compared to experimental results.

First, we measured the ^1^H and ^13^C chemical shifts of cabotegravir in its solid form (microcrystalline powder) via VFMAS SSNMR spectroscopy. The SSNMR spectra have been acquired under 60 kHz MAS on a high-field spectrometer (20.0 T, 850 MHz ^1^H Larmor frequency). As shown in Figure 2a, the ^13^C signals of cabotegravir powders on the ^1^H-^13^C cross-polarization (CP) 1D ^13^C NMR spectrum are well-resolved, indicating that molecules are highly ordered in these microcrystalline samples. To facilitate the ^13^C chemical shift assignment and to probe the hydrogens, we have also recorded a CP-based ^1^H-detected ^1^H-^13^C 2D correlation spectrum (Figure 2b) with a short contact time (200 μs). The ^1^H homodecoupling was not applied under VFMAS during the ^1^H detection, which prevents the bias by chemical shift scaling factors and therefore permits a better direct comparison with those obtained in solution. Even without deuteration, ^1^H signals appear well-resolved on the ^1^H-^13^C 2D NMR spectrum. The full assignment of ^1^H and ^13^C chemical shifts of cabotegravir microcrystalline powders can be found in Figure 2a,b and Tables S5 and S6. In addition, the ^15^N chemical shift assignment was also determined via the ^15^N-^1^H correlations under VFMAS condition (Figure S2), which has been proven to be much more sensitive than the ^15^N-direct detection experiments.

Second, we have screened a scheme of DFT functionals based on the ^1^H and ^13^C chemical shifts obtained via SSNMR experiments. Previously, we have proposed an AF-QM/MM approach for calculating the chemical shifts of molecular crystals [24]. Using this approach, we have tested seven different functionals, namely, B3LYP, B3PW91, M06-2X, M06-L, mPW1PW91, OB98 and OPBE, in ^1^H and ^13^C chemical shift calculations on cabotegravir. The comparison of the experimental and DFT calculated ^1^H and ^13^C chemical shifts of cabotegravir in crystals can be found in Tables S5 and S6. The best performing ^1^H and ^13^C predictions are presented in Figure 2c,d. The overall root mean square error (RMSE) between the predicted ^1^H and ^13^C chemical shifts and the experimental values are visualized in Figure 2e. The mPW1PW91 functional shows the lowest Mean Unsigned Error (MUE) and RMSE (1.6 and 2.1 ppm, respectively) in the calculation of ^13^C chemical shifts, indicating that it is the most accurate one among all tested functionals. The M06-2X, M06-L, OB98 and OPBE functionals exhibit a significantly larger RMSE of ^13^C chemical shifts. These functionals are therefore not suitable for calculating our system. In terms of ^1^H chemical shift prediction, the OB98 and OPBE functionals show satisfying performances. The B3LYP functional shows the best performance on reproducing the experimental ^1^H chemical shifts, with the lowest RMSE of 0.38 ppm. A more detailed analysis of the performance of functionals can be found in Tables S7 and S8. These findings agree well with our previous observations on other structurally distinct organic crystals [24], demonstrating the robustness of our approach. Our screening proposes the optimal functionals B3LYP and mPW1PW91 for ^1^H and ^13^C chemical shift prediction, respectively, which supports the NMR data interpretation in the following section.

### 2.3. The Conformational Changes of Cabotegravir upon Dissolution

We have probed the conformational changes of cabotegravir from solid to solution via chemical shifts. Ideally, a direct comparison of the chemical shifts of this drug as a solid and in aqueous solution would report the structural hotspots involved in such conformational changes. Unfortunately, the extremely low solubility of cabotegravir in water prevents a comprehensive NMR characterization of this drug in aqueous solutions. Therefore, we have taken a two-step approach, which uses DMSO [25,26] as a “broker” solvent as described below, to extract the structural information.

**Figure 3:**
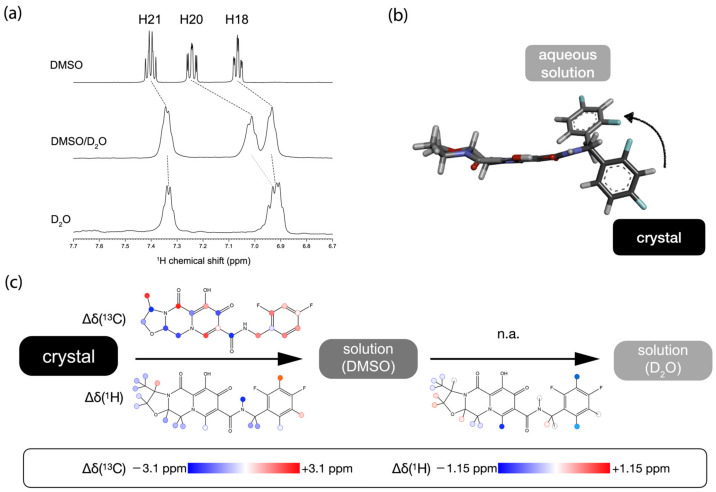
(**a**) ^1^H NMR signals of the difluorobenzene ring undergo significant drifts upon the change of solvent. (**b**) The conformational change of the difluorobenzene ring from the crystal structure to the structural model in aqueous solution. (**c**) Chemical shift changes upon dissolution and solvent switching. The chemical shift changes are shown in a colored heatmap.

First, we mapped the conformational differences of cabotegravir between its solid form and DMSO solution. Cabotegravir can be readily dissolved in DMSO and the ^13^C chemical shift (Figure S3a) and ^1^H (Figure 3a and Figure S3b) of cabotegravir were obtained. As shown in Tables S5 and S6, cabotegravir shows significant overall ^1^H and ^13^C chemical shift changes (RMSE 0.14 and 2.0 ppm, respectively), suggesting that cabotegravir adopts different conformations as a solid and in DMSO solution. Strikingly, the amide proton (H14) undergoes a large (0.7 ppm) downfield chemical shift change passing from solids to DMSO solution (Figure 3c, Tables S6 and S10), indicating the weakening of intramolecular H-bonds at this site upon dissolution. The chemical shift change further propagates to the neighboring C15 methylene and difluorobenzene ring (Figure 3c, Tables S6 and S10). On the other side, significant ^1^H and ^13^C chemical shift changes appear on the fused ring moiety (Figure 3c, Tables S6, S9 and S10). The observed chemical shift changes could arise from the structural differences or the different molecular environment (e.g., polarity) in two distinct states. To delineate these experimental chemical shift changes, we have used the above selected optimal functionals, namely B3LYP and mPW1PW91, for calculating the ^1^H and ^13^C chemical shifts of cabotegravir in solution. We have taken the crystal structure of cabotegravir and calculated the ^1^H and ^13^C chemical shifts of this molecule in implicit DMSO solvent using the DFT approach. Our calculations (Tables S5 and S6) show that the DMSO solvent environment does not induce significant chemical shift changes such as those found experimentally, further supporting the interpretation of observed chemical shift changes as a result of conformational changes.

We then followed the trend of the structural changes of cabotegravir from organic solvent (DMSO) to aqueous solution. For this purpose, an NMR sample of cabotegravir in DMSO/D_2_O (1:1 *v*:*v*) mixed solvent was used first. This NMR sample was prepared by adding water to the DMSO solution of cabotegravir. A large quantity of precipitates formed upon the addition of D_2_O, indicating the significantly reduced drug concentration even in the presence of 50% DMSO. The intensity of the ^1^H NMR signals appeared rather low. Our attempt at ^13^C NMR experiments on this sample using a highly sensitive cryo-probe on a 600 MHz spectrometer was unsuccessful due to the very low drug concentration. Nevertheless, the obtained ^1^H NMR spectrum (Figure 3a and Figure S3c) permitted us to track some key structural changes of cabotegravir in different solvents. The assigned ^1^H chemical shifts are reported in Table S11. Most ^1^H signals, including the methyl protons, experience an upfield drift about 0.08 ppm passing from DMSO to DMSO/D_2_O mixture. The H18 and H20 signals show significantly larger chemical shift changes compared to other signals (Figure 3a), indicating that the difluorobenzene ring is the hotspot of conformational changes in response to the solvent change.

We have further recorded the ^1^H NMR spectrum of cabotegravir in pure D_2_O (Figure S3d). As shown in Figure 3a, The H20 and H21 signals remain at similar positions to those in DMSO/D_2_O mixed solvent. Strikingly, the H20 signal could not be unambiguously identified in the nearby spectral region, indicating a rather large chemical shift change from DMSO/D_2_O solvent to pure water. A tentative candidate of an H20 signal in D_2_O is indicated Figure 3a, and overlaps with the H18 signal. These observations suggest that further conformational changes take place at the difluorobenzene ring. We have also explored the different conformations of cabotegravir by comparing DFT-predicted chemical shifts and the experimental values in water. The optimized C13-N14-C15-C16 and N14-C15-C16-C17 dihedral angles are 48° and 151°, respectively (Figure 3b), at which the difference between the DFT-predicted and experimental ^1^H chemical shifts of H18 and H20 are less than 0.1 ppm and therefore within the RMSE threshold of the DFT method used here. Besides the ^1^H signals on the difluorobenzene ring, several hydrogens on the fused ring moiety, namely H9, also exhibit significant chemical shift changes (Figure 3c). In particular, the large chemical shift change of H9 suggests the overall involvement of the neighboring amide group in the conformational changes.

Our analysis here has identified several chemical moieties as the hotspots involved in the conformational changes upon dissolution (Figure 3c). During the dissolution process, the cabotegravir molecules have to be freed from the crystal lattice via the reorientation of the highly hydrophobic difluorobenzene ring from a hydrophobically stacked channel to a highly polar environment (Figure 3b). This process is likely coupled with the weakening of the intramolecular H-bond formed by the amide linker and the fused ring moiety in solution. These multiple energetically unfavored and coupled events could contribute to the low solubility of cabotegravir, a key pharmaceutical feature needed for its long-acting efficacy.

### 2.4. Multi-Tasking Pharmacophores in Cabotegravir

**Figure 4:**
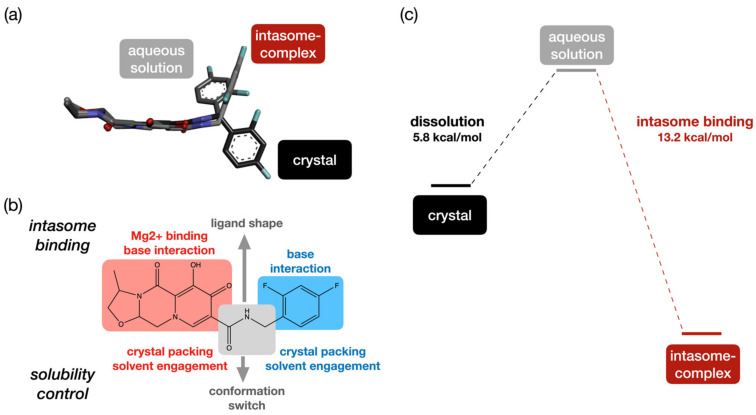
(**a**) Comparison of cabotegravir crystal structure with the best calculated structural model in aqueous solution and the structural model of the intasome bound state. (**b**) Multitasking pharmacophores in cabotegravir. The fused ring moiety (red), middle linker (gray) and difluorobenzene ring (blue) all participate in both the determination of water solubility and HIV intasome binding. (**c**) The Gibbs free energies of the dissolution and intasome binding of cabotegravir. The values were calculated using the reported solubility [11] and IC50 [12] of this drug. These experimentally reported equilibrium constants have been converted to Gibbs free energy using the standing equation ΔG = −RTlnK.

The participation of multiple moieties in the conformational changes depicts a rather complex mechanism underlying the low water solubility of cabotegravir (Figure 4b). All of these solubility-determining moieties are also required for high anti-HIV activities (Figure 4b). The SAR of these pharmacophores in cabotegravir-family drugs has already been investigated using medicinal chemical [12] and structural biology approaches [13]. A medicinal chemistry campaign [12] has shown that the fluorine substitution of the benzene ring and the structure of the oxazoline ring moiety could be varied without compromising the anti-HIV activity of the molecule. A number of structures of cabotegravir analogues in complex with intasome have been determined [13]. By combining the SAR and dissolution-related conformational changes of various chemical moieties in cabotegravir, a picture of multitasking pharmacophores in this long-acting HIV PrEP drug emerges (Figure 4b).

First, in the dolutegravir—intasome complex structure, the fused ring moiety acts as a platform for accommodating the peripheral oxygens that coordinate with magnesium in the intasome [13]. The pyridine rings are also stacked with a base of nucleotides [13]. The rather flat boat-like conformation of the neighboring piperazine ring is required for permitting the intermolecular pyridine–base stacking. This conformation of the piperazine ring also orients the neighboring fused isomorpholine ring away from the target nucleotide base, therefore preventing a steric clashing [24]. In the case of cabotegravir, the isomorpholine ring is replaced by a smaller oxazole ring (Figure S4), which keeps the whole fused ring moiety flatter than that in dolutegravir. This molecular feature permits similar intermolecular interactions with the intasome, while allowing the cabotegravir molecule to be better packed in its crystal lattice. The fused ring moiety in cabotegravir seems, therefore, better optimized for both biological and physicochemical properties.

Second, the difluorobenzene ring stacks with another base in the intasome. As mentioned above, this moiety is intimately involved in solvent-related conformational changes (Figure 4a), which is crucial for the low water solubility. The fluorine substitutions on this ring set the van der Waals interactions in the crystals, while remaining tolerable for the interaction with the intasome. Therefore, this pharmacophore is dual functional as well, which balances the anti-virus activity and the solubility.

Third, the middle linker permits the reorientation of ring moieties on two sides of the molecule in different molecular environments (Figure 4b). This linker permits both a sheet structure formation of the fused ring and the stacking of the difluorobenzene rings in crystals via both intra- and intermolecular H-bonds. In solution, the intramolecular H-bond that locks the amide linker could be weakened, and this is coupled with a conformational change on the neighboring ring moieties. In the intasome, the distinct conformation of this linker permits the reorientation of the fused ring moiety for interactions with ions and the base in the intasome (Figure 4a and Figure S4). Here, the amide group acts as a switch for controlling the molecular packing, solvent engagement and docking conformation for intasome binding, thanks to its capacity for forming intra- and intermolecular H-bonds that could be modified upon dissolution.

In summary, multiple pharmacophores in cabotegravir show unique structural features that are required for both low water solubility and high antiviral activity. A sophisticated molecular design permits the proper energetics of its dissolution and intasome binding (Figure 4c). In cabotegravir, these multitasking pharmacophores set a balance between the water solubility and target-binding affinity that are required for long-acting HIV PrEP.

## 3. Methods

### 3.1. The Crystallization and XRD Structural Determination of Cabotegravir

Solvent evaporation technique was used to grow the cabotegravir sample and XRD to detect the crystal structures of cabotegravir. Cabotegravir (5 mg, Aldrich-Sigma, St. Louis, MI, USA) was dissolved in 400 μL dichloromethane and was incubated at room temperature via solvent volatilization. After four days, colorless crystals of cabotegravir with a rod shape were obtained. A Bruker D8 venture diffractometer was used to collect diffraction intensity data, with CuK_α_ radiation used as a light source and the scanning mode set as φ/ω. We collected a total of 17,089 diffraction points, 3165 independent diffraction points, and eventually obtained 3114 observable points (I/sigma ≥ 2.0). To identify the atomic species and obtain the positions of 29 non-hydrogen atoms of the cabotegravir crystal, the structure was determined via the SHELXS97 program [27]. The least square method was used to modify structural parameters and identify atomic species. The positions of all hydrogen atoms were obtained by geometric calculation. The solved cabotegravir structure is deposited under the CCDC number 2034742.

### 3.2. SSNMR Spectroscopy

Cabotegravir powder (ca. 3 mg) was packed into a 1.3 mm ZrO_2_ rotor. The ^1^H-^13^C 1D CP and the 2D CP-based ^1^H-^13^C/^15^N HSQC spectra were acquired using a Bruker Avance III NMR spectrometer (Bruker, Billerica, MA, USA) operating at 850 MHz (^1^H Larmor frequency). All the spectra were acquired under the VF MAS (60 kHz) condition. These ^1^H direct detection experiments without ^1^H decoupling during the acquisition permit the determination of ^1^H chemical shifts without bias by the off-set dependent ^1^H chemical shifts scaling factors. Our initial efforts in acquiring the natural abundance ^15^N spectra using the ^15^N direct detection scheme under low MAS was unsuccessful due to the low sensitivity. The ^1^H-detected ^15^N-^1^H 2D experiment offers a practical solution for detecting the ^15^N signals of our sample. Low-power double-quantum (DQ) ^1^H-^13^C/^15^N CP was applied for the ^1^H-^13^C/^15^N and ^13^C/^15^N-^1^H magnetization transfers. The CP contact times were set to 200 μs and 10 ms for the ^1^H-^13^C and ^1^H-^15^N magnetization transfers, respectively. Low-power heterodecoupling (WALTZ16 [28] for ^13^C and GARP [29] for ^15^N) was applied during the ^1^H acquisition period. The ^13^C-^1^H HSQC spectrum was acquired with 2560 (F2) and 96 (F1) points and was processed with a 4096 (F2) * 512 (F1) matrix. QSine window function (SSB = 2) was applied for both F2 and F1 dimensions. The ^15^N-^1^H HSQC spectrum was acquired with 2560 (F2) and 14 (F1) points and was processed with a 4096 (F2) * 128 (F1) matrix. QSine window function (SSB = 4) was applied for both F2 and F1 dimensions. All ^13^C chemical shifts were referenced indirectly to TMS using an adamantane (38.48 and 29.46 ppm as referenced to TMS) or alanine (177.84 ppm as referenced to TMS) sample as the intermediate external reference. The ^1^H and ^15^N chemical shifts calibrations were carried out by converting the referenced ^13^C spectrometer frequency to ^1^H or ^15^N spectrometer frequencies via the gyromagnetic scaling factor (0.251449530 and 0.402979940 for ^13^C/^1^H and ^15^N/^13^C, respectively).

### 3.3. Solution NMR Spectroscopy

The ^1^H NMR spectra of GSK were acquired at 298 K using a Bruker AVANCE III 600 MHz spectrometer (Bruker, Billerica, MA, USA) equipped with a 5 mm diameter ^1^H/^13^C/^15^N TCI Cryoprobe. The pulse program noesygppr1d from the Bruker pulse sequence library was used. The ^13^C 1D spectrum was acquired with the standard pulse program. ^1^H NMR spectra were recorded with the 128 scans and 128 K data points. The ^13^C NMR spectrum of DMSO solution was recorded with 8192 scans and transients with 8192 and 64K data points. For the ^13^C NMR spectra in DMSO/D_2_O mixed solvent, an attempt of 16K scans was carried out, while no signal from cabotegravir was visible.

### 3.4. The AF-QM/MM Method for Chemical Shift Calculations

In the automated fragmentation quantum mechanics/molecular mechanics (AF-QM/MM) approach, a 5 × 5 × 5 supercell is used to calculate the isotropic chemical shifts of ^1^H, ^13^C and ^15^N in molecular crystals [30,31,32,33,34]. In this method, a single molecule is selected as the core region, and other molecules within a certain distance from the core region are regarded as the buffer region. The definition criteria of the buffer region are as follows: (1) the distance between any atom in the molecule outside the core region and any atom in the core region is less than 3.5 Å (at least one is a non-hydrogen atom), and (2) the distance between the H atom on the molecule in the core region and the H atom of the molecule outside the core region is less than 2.5 Å. Any molecules that meet the above criteria are considered as buffer regions. Both the core and buffer regions are described by quantum mechanics (QM), and the rest of the molecules are described by molecular mechanics (MM). For the MM region, the point charge model is used to consider the electrostatic effect of the background charge, and the AM1-BCC charge model is used to describe the embedding field in the MM region [35].

The size of each molecular fragment is independent of the total crystal size. Each fragment-based AF-QM/MM calculation only contains a limited number of molecules near the central molecule in the QM region, and thus the method is linear scaling and trivially parallel. For each fragment, NMR calculations are performed with the GIAO [36,37] method using the Gaussian16 package [38]. The calculated chemical shifts are referenced to the ^1^H, ^13^C isotropic shielding constants of tetramethylsilane (TMS) and the ^15^N isotropic shielding constant of ammonia computed at the same level in the gas phase (B3LYP/6-31G**: ^1^H 31.75 ppm, ^13^C 191.74 ppm, ^15^N 262.56 ppm; B3PW91/6-31G**: ^1^H 31.70 ppm, ^13^C 194.87 ppm, ^15^N 265.00 ppm; mPW1PW91/6-31G**: ^1^H 31.75 ppm, ^13^C 196.21 ppm, ^15^N 266.52 ppm). In addition, the molecular crystals in the reference group were optimized using the Vienna Ab initio Simulation Package (VASP) [39] before NMR chemical shift calculations. The Perdew–Burke–Ernzerhof form of the Generalized Gradient Approximation functional (GGA-PBE) was used to obtain the exchange and correlation terms [40]. The D3 dispersion correction has been applied to describe the dispersion between molecules [41]. The initial structure was relaxed using a convergence criterion of 10^−5^ eV for energy and 0.01 eV/Å for atomic forces. The valence electrons were described by setting a cut-off energy of 340 eV using the plain wave basis set.

### 3.5. The Structure Modeling of Cabotegravir in Different Environments

We used Gaussview 5.0 [42] to generate various conformations of cabotegravir by exploring the dihedral angle between the difluorobenzene ring and the amide moiety. The C13-N14-C15-C16 and N14-C15-C16-C17 dihedral angles were adjusted in steps of 60°. The initial dihedral angles for C13-N14-C15-C16 and N14-C15-C16-C17 dihedral angles are −101.6° and −149.3°, respectively. The ^1^H chemical shifts of the difluorobenzene ring in the generated structures were calculated in water and DMSO solvents and matched with the experimental values for selecting the best structural model. For modeling the structure of intasome-bound cabotegravir, cabotegravir structure solved by X-ray crystallography in our work was first adjusted to match the conformation of the dolutegravir molecule in the experimental intasome-dolutegravir complex (PDB 3S3M).

## 4. Conclusions

The water solubility of drugs is a fundamentally important and yet challenging to study physicochemical character. In the case of cabotegravir, a highly successful anti-HIV drug, the low solubility is pivotal to its success in long-acting PrEP, a new paradigm of infectious disease control. The combination of an array of highly complementary structural characterization and computational chemistry techniques, namely XRD, SSNMR, solution NMR, DFT and AF-QM/MM methods, has permitted the unravelling of the molecular features underlying this unique character of cabotegravir. Our work provides the first crystal structure of this family of long-acting HIV PrEP drugs. Our study shows that the pharmacophores in cabotegravir are multitasking, taking various roles in balancing the anti-HIV activity and the water solubility by controlling the crystal packing, molecular conformations in solution and intasome interaction. The new molecular insights into cabotegravir that are provided by this work will support the further engineering of long-acting HIV PrEP agents, for example to guide the structural optimization for even lower drug solubility, which is required for longer lasting HIV PrEP, without compromising the anti-HIV activity.

Our current work bears several main limitations. First, the ^13^C NMR data on cabotegravir in water was not obtained due to the very low solubility. Second, our work has focused on the conventional crystal structure of cabotegravir rather than the structures of its nano-particle formulation, which is the form used in real-life HIV PrEP. Third, our work has not yielded a quantitative analysis of each pharmacophore in the solubility determination. The further implementation of advanced NMR approaches as well as the development of computational methods are required for covering these gaps.

## Figures and Tables

**Table 1:** The crystal information and XRD refinement parameters for cabotegravir.

Identification Code	Cabotegravir
Empirical formula	C_19_H_17_F_2_N_3_O_5_
Formula weight	405.35 g/mol
Temperature	182(2) K
Wavelength	1.54178 Å
Crystal system, space group	Orthorhombic, P2_1_2_1_2_1_
Unit cell dimensions	a = 7.3002(5) Å alpha = 90 deg.
	b = 7.3002(1) Å beta = 90 deg.
	c = 32.343(2) Å gamma = 90 deg.
Volume	1723.63(16) Å^3^
Z, Calculated density	4, 1.562 Mg/m^3^
Absorption coefficient	1.103 mm^−1^
F(000)	840
Crystal size	0.260 × 0.140 × 0.080 mm
Theta range for data collection	5.471 to 68.809 deg.
Limiting indices	−8 ≤ h ≤ 8, −8 ≤ k ≤ 8, −31 ≤ l ≤ 39
Reflections collected/unique	17,089/3165 [R(int) = 0.0349]
Completeness to theta = 67.679	99.4%
Absorption correction	Semi-empirical from equivalents
Max. and min. transmission	0.7531 and 0.6369
Refinement method	Full-matrix least-squares on *F*^2^
Data/restraints/parameters	3165/0/262
Goodness-of-fit on *F*^2^	1.059
Final R indices [I > 2sigma(I)]	R_1_ = 0.0274, wR_2_ = 0.0722
R indices (all data)	R_1_ = 0.0279, wR_2_ = 0.0730
Absolute structure parameter	0.01(4)
Extinction coefficient	n/a
Largest diff. peak and hole	0.157 and −0.172 e·A^−3^

## Data Availability

The data generated by this work can be obtained directly from the authors upon reasonable requests.

## Supplementary Materials

The following supporting information can be downloaded at: https://www.mdpi.com/article/10.3390/molecules29020376/s1. The measured atomic coordinates, bond lengths, angles, torsional angles and hydrogen bonds for the cabotegravir crystal, experimental and DFT calculated ^1^H and ^13^C chemical shifts, additional NMR spectra and structural comparisons can be found in supporting information.
