# Supplementary material for: Multitasking Pharmacophores Support Cabotegravir-Based Long-Acting HIV Pre-Exposure Prophylaxis (PrEP)
Source: Molecules. 2024 Jan 11;29(2):376. doi: 10.3390/molecules29020376 (PMC10819392; doi:10.3390/molecules29020376)

Supporting Information for
Multitasking pharmacophores support cabotegravir-based long-acting HIV Pre-Exposure Prophylaxis (PrEP)

Zheng Wan[1], Man Shi[1], Yanqing Gong[2], Massimo Lucci[3], Jinjin Li[4], Jiahai Zhou[5], Xiao-Liang Yang*[6], Moreno Lelli[3,7*], Xiao He[1,8*], Jiafei Mao[9,10*]

[1] Shanghai Engineering Research Center of Molecular Therapeutics and New Drug Development, Shanghai Frontiers Science Center of Molecule Intelligent Syntheses, School of Chemistry and Molecular Engineering, East China Normal University, Shanghai, 200062, China

[2] State Key Laboratory of New Drug and Pharmaceutical Process, Shanghai Institute of Pharmaceutical Industry, China State Institute of Pharmaceutical Industry, 285 Gebaini Road, Shanghai, 201203, China

[3] C.I.R.M.M.P. - Consorzio Interuniversitario Risonanze Magnetiche di Metallo Proteine, Via L. Sacconi 6, 50019 Sesto Fiorentino, Firenze, Italy

[4] Key Laboratory for Thin Film and Microfabrication of Ministry of Education, Department of Micro/Nano-electronics, Shanghai Jiao Tong University, Shanghai, 200240, China

[5] CAS Key Laboratory of Quantitative Engineering Biology, Shenzhen Institute of Synthetic Biology, Shenzhen Institute of Advanced Technology, Chinese Academy of Sciences, Shenzhen, 518055, China

6 State Key Laboratory of Coordination Chemistry and Jiangsu Key Laboratory of Advanced Organic Materials, School of Chemistry and Chemical Engineering, Nanjing University, Nanjing, 210023, China

[7] Department of Chemistry "Ugo Schiff", Magnetic Resonance Center (CERM) University of Florence, Via della Lastruccia 3, 50019 Sesto Fiorentino, Florence, Italy

[8] New York University–East China Normal University Center for Computational Chemistry, New York University Shanghai, Shanghai, 200062, China

[9] Beijing National Laboratory for Molecular Sciences (BNLMS), Institute of Chemistry, Chinese Academy of Sciences, Zhongguancun North First Street 2, 100190 Beijing, China

[10] Center for Physicochemical Analysis and Measurement, Institute of Chemistry, Chinese Academy of Science, Zhongguancun North First Street 2, 100190 Beijing, China

*Correspondence to:

Jiafei Mao (jmao2023@iccas.ac.cn),

Xiao He (xiaohe@phy.ecnu.edu.cn),

Moreno Lelli (moreno.lelli@unifi.it),

Xiaoliang Yang (yxlnmr@nju.edu.cn)

Table S1 The atomic coordinates (10^{-4} Å) and equivalent isotropic displacements (10^{-3} Å^2) of GSK744 crystal.

**Atom**	**x**	**y**	**z**	**U(eq)**
F(1)	164(2)	-392(2)	5119(1)	44(1)
F(2)	-42(3)	5755(2)	5510(1)	57(1)
O(1)	-1161(2)	1631(2)	3527(1)	34(1)
O(2)	4639(2)	1466(2)	3560(1)	27(1)
O(3)	6588(2)	1690(2)	2881(1)	29(1)
O(4)	6537(2)	2725(2)	2062(1)	27(1)
O(5)	1411(2)	1559(2)	1347(1)	31(1)
N(1)	1344(2)	901(2)	3904(1)	26(1)
N(2)	1995(2)	2090(2)	2462(1)	22(1)
N(3)	4014(2)	1581(2)	1738(1)	23(1)
C(1)	241(2)	1844(3)	4596(1)	25(1)
C(2)	164(3)	1402(3)	5012(1)	28(1)
C(3)	88(3)	2672(3)	5326(1)	34(1)
C(4)	71(3)	4470(3)	5208(1)	34(1)
C(5)	151(3)	5022(3)	4802(1)	34(1)
C(6)	238(3)	3691(3)	4499(1)	28(1)
C(7)	306(3)	366(3)	4268(1)	30(1)
C(8)	523(3)	1441(3)	3554(1)	23(1)
C(9)	1775(2)	1742(2)	3194(1)	22(1)
C(10)	3732(3)	1673(2)	3232(1)	22(1)
C(11)	4756(3)	1839(2)	2848(1)	22(1)
C(12)	3908(2)	2040(3)	2474(1)	21(1)
C(13)	4951(3)	2162(2)	2073(1)	22(1)
C(14)	4603(3)	1931(3)	1307(1)	24(1)
C(15)	2939(3)	1167(3)	1080(1)	31(1)
C(16)	2039(3)	1151(3)	1749(1)	23(1)
C(17)	1089(3)	2367(3)	2059(1)	24(1)
C(18)	1001(3)	1959(3)	2810(1)	22(1)
C(19)	6398(3)	1031(3)	1185(1)	31(1)
H(3A)	6887	1716	3132	44
H(1A)	2549	869	3913	31
H(3B)	49	2318	5608	41
H(5A)	148	6286	4731	40
H(6A)	297	4052	4217	34
H(7A)	863	-750	4389	35
H(7B)	-961	58	4184	35
H(14A)	4686	3282	1259	29
H(15A)	2784	1773	809	37
H(15B)	3067	-169	1036	37
H(16A)	1835	-171	1816	28
H(17A)	1185	3666	1974	29
H(17B)	-225	2041	2079	29
H(18A)	-296	2019	2790	27
H(19A)	6683	1333	897	46
H(19B)	7382	1481	1364	46
H(19C)	6289	-300	1215	46

Table S2 The measured bond lengths (Å) and angles (degree) for the GSK744 crystal.

Atoms	Bond length	Atoms	Bond length
F(1)-C(2)	1.355(2)	C(5)-C(6)	1.381(3)
F(2)-C(4)	1.357(2)	C(5)-H(5A)	0.9500
O(1)-C(8)	1.240(2)	C(6)-H(6A)	0.9500
O(2)-C(10)	1.260(2)	C(7)-H(7A)	0.9900
O(3)-C(11)	1.346(2)	C(7)-H(7B)	0.9900
O(3)-H(3A)	0.8400	C(8)-C(9)	1.495(2)
O(4)-C(13)	1.229(2)	C(9)-C(18)	1.374(2)
O(5)-C(16)	1.409(2)	C(9)-C(10)	1.435(3)
O(5)-C(15)	1.440(2)	C(10)-C(11)	1.454(3)
N(1)-C(8)	1.340(2)	C(11)-C(12)	1.365(3)
N(1)-C(7)	1.455(2)	C(12)-C(13)	1.507(3)
N(1)-H(1A)	0.8800	C(14)-C(19)	1.518(3)
N(2)-C(18)	1.342(2)	C(14)-C(15)	1.525(3)
N(2)-C(12)	1.397(2)	C(14)-H(14A)	1.0000
N(2)-C(17)	1.476(2)	C(15)-H(15A)	0.9900
N(3)-C(13)	1.350(2)	C(15)-H(15B)	0.9900
N(3)-C(16)	1.476(2)	C(16)-C(17)	1.509(3)
N(3)-C(14)	1.481(2)	C(16)-H(16A)	1.0000
C(1)-C(6)	1.384(3)	C(17)-H(17A)	0.9900
C(1)-C(2)	1.385(3)	C(17)-H(17B)	0.9900
C(1)-C(7)	1.513(3)	C(18)-H(18A)	0.9500
C(2)-C(3)	1.375(3)	C(19)-H(19A)	0.9800
C(3)-C(4)	1.367(3)	C(19)-H(19B)	0.9800
C(3)-H(3B)	0.9500	C(19)-H(19C)	0.9800
C(4)-C(5)	1.376(3)		
**Atoms**	**Angle**	**Atoms**	**Angle**
C(11)-O(3)-H(3A)	109.5	C(3)-C(2)-C(1)	124.17(19)
C(16)-O(5)-C(15)	105.01(14)	C(4)-C(3)-C(2)	116.26(18)
C(8)-N(1)-C(7)	122.03(17)	C(4)-C(3)-H(3B)	121.9
C(8)-N(1)-H(1A)	119.0	C(2)-C(3)-H(3B)	121.9
C(7)-N(1)-H(1A)	119.0	F(2)-C(4)-C(3)	117.69(19)
C(18)-N(2)-C(12)	121.00(15)	F(2)-C(4)-C(5)	119.2(2)
C(18)-N(2)-C(17)	120.50(15)	C(3)-C(4)-C(5)	123.14(19)
C(12)-N(2)-C(17)	118.45(15)	C(4)-C(5)-C(6)	118.23(19)
C(13)-N(3)-C(16)	122.86(15)	C(4)-C(5)-H(5A)	120.9
C(13)-N(3)-C(14)	123.66(15)	C(6)-C(5)-H(5A)	120.9
C(16)-N(3)-C(14)	110.05(14)	C(5)-C(6)-C(1)	121.68(18)
C(6)-C(1)-C(2)	116.53(18)	C(5)-C(6)-H(6A)	119.2
C(6)-C(1)-C(7)	122.45(17)	C(1)-C(6)-H(6A)	119.2
C(2)-C(1)-C(7)	121.02(18)	N(1)-C(7)-C(1)	113.07(16)
F(1)-C(2)-C(3)	117.55(17)	N(1)-C(7)-H(7A)	109.0
F(1)-C(2)-C(1)	118.28(17)	C(1)-C(7)-H(7A)	109.0
N(1)-C(7)-H(7B)	109.0	O(5)-C(15)-C(14)	104.82(14)
C(1)-C(7)H(7B)	109.0	O(5)-C(15)-H(15A)	110.8
H(7A)-C(7)-H(7B)	107.8	C(14)-C(15)-H(15A)	110.8
O(1)-C(8)-N(1)	122.36(17)	O(5)-C(15)-H(15B)	110.8
O(1)-C(8)-C(9)	122.38(16)	C(14)-C(15)-H(15B)	110.8
N(1)-C(8)-C(9)	115.22(16)	H(15A)-C(15)-H(15B)	108.9

C(18)-C(9)-C(10)	119.29(16)	O(5)-C(16)-N(3)	104.53(14)
C(18)-C(9)-C(8)	117.93(16)	O(5)-C(16)C(17)	109.83(15)
C(10)-C(9)-C(8)	122.61(16)	N(3)-C(16)-C(17)	109.84(15)
O(2)-C(10)-C(9)	126.72(17)	O(5)-C(16)-H(16A)	110.8
O(2)-C(10)-C(11)	117.33(16)	N(3)-C(16)-H(16A)	110.8
C(9)-C(10)-C(11)	115.94(16)	C(17)-C(16)-H(16A)	110.8
O(3)-C(11)-C(12)	121.99(16)	N(2)-C(17)-C(16)	107.51(15)
O(3)-C(11)-C(10)	115.84(15)	N(2)-C(17)-H(17A)	110.2
C(12)-C(11)-C(10)	122.09(17)	C(16)-C(17)-H(17A)	110.2
C(11)-C(12)-N(2)	118.73(16)	N(2)-C(17)-H(17B)	110.2
C(11)-C(12)-C(13)	122.64(17)	C(16)-C(17)H(17B)	110.2
N(2)-C(12)-C(13)	118.62(16)	H(17A)-C(17)-H(17B)	108.5
O(4)-C(13)-N(3)	123.93(17)	N(2)-C(18)-C(9)	122.95(17)
O(4)-C(13)-C(12)	121.41(16)	N(2)-C(18)-H(18A)	118.5
N(3)-C(13)-C(12)	114.65(16)	C(9)-C(18)-H(18A)	118.5
N(3)-C(14)-C(19)	114.89(15)	C(14)-C(19)-H(19A)	109.5
N(3)-C(14)-C(15)	99.11(14)	C(14)-C(19)-H(19B)	109.5
C(19)-C(14)-H(14A)	113.85(16)	H(19A)-C(19)-H(19B)	109.5
N(3)-C(14)H(14A)	109.5	C(14)-C(19)-H(19C)	109.5
C(19)-C(14)-H(14A)	109.5	H(19A)-C(19)-H(19C)	109.5
C(15)-C(14)-H(14A)	109.5	H(19B)-C(19)-H(19C)	109.5

Table S3 The measured torsional angles [degree] for the GSK744 crystal.

C(6)-C(1)-C(2)-F(1)	180.00(19)
C(7)-C(1)-C(2)-F(1)	0.6(3)
C(6)-C(1)-C(2)-C(3)	-0.1(3)
C(7)-C(1)-C(2)-C(3)	-179.5(2)
F(1)-C(2)-C(3)-C(4)	-179.4(2)
C(1)-C(2)-C(3)-C(4)	0.7(4)
C(2)-C(3)-C(4)-F(2)	178.7(2)
C(2)-C(3)-C(4)-C(5)	-0.9(4)
F(2)-C(4)-C(5)-C(6)	-179.0(2)
C(3)-C(4)-C(5)-C(6)	0.5(4)
C(4)-C(5)-C(6)-C(1)	0.1(4)
C(2)-C(1)-C(6)-C(5)	-0.3(3)
C(7)-C(1)-C(6)-C(5)	179.0(2)
C(8)-N(1)-C(7)-C(1)	-101.8(2)
C(6)-C(1)-C(7)-N(1)	32.6(3)
C(2)-C(1)-C(7)-N(1)	-148.08(19)
C(7)-N(1)-C(8)-O(1)	4.7(3)
C(7)-N(1)-C(8)-C(9)	-173.43(16)
O(1)-C(8)-C(9)-C(18)	-10.1(3)
N(1)-C(8)-C(9)-C(18)	168.05(17)
O(1)-C(8)-C(9)-C(10)	174.70(19)
N(1)-C(8)-C(9)-C(10)	-7.2(3)
C(18)-C(9)-C(10)-O(2)	-179.37(18)
C(8)-C(9)-C(10)-O(2)	-4.2(3)
C(18)-C(9)-C(10)-C(11)	-0.2(3)
C(8)-C(9)-C(10)-C(11)	174.96(16)
O(2)-C(10)-C(11)-O(3)	2.2(2)
C(9)-C(10)-C(11)-O(3)	-177.07(16)
O(2)-C(10)-C(11)-C(12)	179.00(18)
C(9)-C(10)-C(11)-C(12)	-0.3(3)
O(3)-C(11)-C(12)-N(2)	176.70(17)
C(10)-C(11)-C(12)-N(2)	0.1(3)
O(3)-C(11)-C(12)-C(13)	-1.7(3)
C(10)-C(11)-C(12)-C(13)	-178.30(16)
C(18)-N(2)-C(12)-C(11)	0.6(3)
C(17)-N(2)-C(12)-C(11)	178.06(16)
C(18)-N(2)-C(12)-C(13)	179.01(16)
C(17)-N(2)-C(12)-C(13)	-3.5(2)
C(16)-N(3)-C(13)-O(4)	-171.40(17)
C(14)-N(3)-C(13)-O(4)	-14.4(3)
C(16)-N(3)-C(13)-C(12)	9.7(3)
C(14)-N(3)-C(13)-C(12)	166.71(16)
C(11)-C(12)-C(13)-O(4)	-26.0(3)
N(2)-C(12)-C(13)-O(4)	155.58(18)
C(11)-C(12)-C(13)-N(3)	152.89(18)
N(2)-C(12)-C(13)-N(3)	-25.5(2)
C(13)-N(3)-C(14)-C(19)	64.4(2)
C(16)-N(3)-C(14)-C(19)	-136.01(17)
C(13)-N(3)-C(14)-C(15)	-173.83(18)
C(16)-N(3)-C(14)-C(15)	-14.28(19)

C(16)-O(5)-C(15)-C(14)	-42.22(19)
N(3)-C(14)-C(15)-O(5)	33.47(18)
C(19)-C(14)-C(15)-O(5)	155.95(16)
C(15)-O(5)-C(16)-N(3)	31.90(19)
C(15)-O(5)-C(16)-C(17)	149.68(16)
C(13)-N(3)-C(16)-O(5)	149.70(17)
C(14)-N(3)-C(16)-O(5)	-10.0(2)
C(13)-N(3)-C(16)-C(17)	31.9(2)
C(14)-N(3)-C(16)-C(17)	-127.83(16)
C(18)-N(2)-C(17)-C(16)	-138.09(17)
C(12)-N(2)-C(17)-C(16)	44.4(2)
O(5)-C(16)-C(17)-N(2)	-171.32(14)
N(3)-C(16)-C(17)-N(2)	-56.88(19)
C(12)-N(2)-C(18)-C(9)	-1.0(3)
C(17)-N(2)-C(18)-C(9)	-178.51(17)
C(10)-C(9)-C(18)-N(2)	0.8(3)

Table S4 The measured hydrogen bonds for the GSK744 crystal.

**D-H...A**	**d(D-H) (Å)**	**d(H...A) (Å)**	**d(D...A) (Å)**	**<(DHA) (degree)**
O(3)-H(3A)...O(1)	0.84	1.92	2.6587(18)	146.8
O(3)-H(3A)...O(2)	0.84	2.16	2.6215(18)	114.9
N(1)-H(1A)...O(2)	0.88	1.96	2.682(2)	138.8

Table S5 The comparison of calculated and observed chemical shifts for ^{13}C atoms. All values are in ppm.

Atom	Chemical shift (crystal, SSNMR)	Chemical shift (crystal, AF-QM/MM)	Chemical shift (solution, NMR)	Chemical shift (solution, DFT)
**C2**	74.5	76.5	72.4	74.5
**C3**	56.1	57.1	52.9	57.0
**C5**	155.8	154.2	158.9	155.3
**C6**	152.0	152.0	153.1	149.0
**C7**	172.6	170.5	170.4	168.1
**C8**	114.8	117.1	114.9	114.7
**C9**	139.2	137.7	141.5	139.2
**C10a**	118.9	119.9	117.4	118.8
**C11**	53.7	58.1	51.4	59.0
**C11a**	84.0	85.9	81.7	86.6
**C12**	14.0	17.5	16.6	16.7
**C13**	166.7	165.1	163.6	163.8
**C15**	34.3	39.7	35.8	38.6
**C16**	122.8	123.2	122.4	123.2
**C17**	158.6	157.6	160.2	158.9
**C18**	103.2	104.3	103.8	102.9
**C19**	161.4	160.2	161.5	160.6
**C20**	110.2	109.2	111.4	109.9
**C21**	129.3	127.8	130.8	127.9

Table S6 The comparison of calculated and observed chemical shifts for ^{1}H atoms. All values are in ppm.

Atom	Chemical shift (crystal, SSNMR)	Chemical shift (crystal, AF-QM/MM)	Chemical shift (solution, NMR)	Chemical shift (solution, DFT)
**H2**	4.20	4.27	4.03 (3.66,4.4)	4.28
**H3**	3.97	4.88	4.30	4.32
**H9**	8.80	8.90	8.47	8.34
**H11**	4.76	4.37	4.45 (4.01,4.89)	4.32
**H11a**	5.79	5.82	5.39	5.62
**H12**	1.64	1.66	1.35	1.68
**H14**	11.45	12.14	10.30	11.08
**H15**	4.99	5.22	4.55	4.84
**H18**	6.73	6.74	7.07	7.22
**H20**	7.04	7.33	7.24	7.29
**H21**	7.46	7.90	7.40	7.80

Table S7 The performance of AF-QM/MM calculated ^{13}C chemical shifts for the carbon set using different density functionals with the 6-31G** basis set as compared to experimental results. MaxD denotes the maximum deviation. All values are in ppm.

Density functional	RMSE	MUE	MaxD	Ref.
**B3LYP**	2.6	2.3	4.2	191.7
**B3PW91**	2.4	2.0	5.0	194.9
**mPW1PW91**	2.1	1.6	5.1	196.2
**M06-L**	8.7	8.3	14.6	193.7
**M06-2X**	11.5	10.5	15.4	196.7
**OB98**	7.2	6.3	13.3	192.0
**OPBE**	7.3	6.1	13.4	195.5

Table S8 The performance of AF-QM/MM calculated ^{1}H chemical shifts for the carbon set using different density functionals with the 6-31G** basis set as compared to experimental results. MaxD denotes the maximum deviation. All values are in ppm.

Density functional	RMSE	MUE	MaxD	Ref.
**B3LYP**	0.38	0.26	0.85	31.75
**B3PW91**	0.41	0.30	0.83	31.70
**mPW1PW91**	0.44	0.34	0.86	31.75
**M06-L**	0.83	0.74	1.39	31.97
**M06-2X**	0.94	0.81	1.90	31.87
**OB98**	0.38	0.31	0.73	31.64
**OPBE**	0.38	0.33	0.62	31.53

Table S9 Comparison of ^{13}C chemical shifts between solid-state and solution samples and between experiments and DFT calculations at the mPW1PW91/6-31G** level, and considering the Influence of DMSO Solvent Effects to Ensure Consistency with Experimental Conditions. The chemical shift differences are in ppm.

**Atom**	**Δδ(^{13}C) (solution/exp vs. crystal/exp)**	**Δδ(^{13}C) (crystal/exp vs. crystal/calc)**	**Δδ(^{13}C) (solution/exp vs. solution/calc)**	**Δδ(^{13}C) (solution/calc vs. crystal/calc)**
**C2**	-2.1	-2	-2.1	-2.0
**C3**	-3.2	-1	-4.1	-0.1
**C5**	3.1	1.6	3.6	1.0
**C6**	1.1	0	4.1	-3.0
**C7**	-2.2	2.1	2.3	-2.4
**C8**	0.1	-2.3	0.2	-2.5
**C9**	2.3	1.5	2.3	1.4
**C10a**	-1.5	-1	-1.4	-1.1
**C11**	-2.3	-4.4	-7.6	0.9
**C11a**	-2.3	-1.9	-4.9	0.7
**C12**	2.6	-3.5	-0.1	-0.7
**C13**	-3.1	1.6	-0.2	-1.3
**C15**	1.5	-5.4	-2.8	-1.1
**C16**	-0.4	-0.4	-0.8	0.0
**C17**	1.6	1	1.3	1.3
**C18**	0.6	-1.1	0.9	-1.4
**C19**	0.1	1.2	0.9	0.4
**C20**	1.2	1	1.5	0.7
**C21**	1.5	1.5	2.9	0.1

Table S10 Comparison of ^{1}H chemical shifts between solid-state and solution samples and between experiments and DFT calculations at the B3LYP/6-31G** level, and considering the Influence of DMSO Solvent Effects to Ensure Consistency with Experimental Conditions. The chemical shift differences are in ppm.

Atom	Δδ(^{1}H) (solution/exp vs. crystal/exp)	Δδ(^{1}H) (crystal/exp vs. crystal/calc)	Δδ(^{1}H) (solution/exp vs. solution/calc)	Δδ(^{1}H) (solution/calc vs. crystal/calc)
**H2**	-0.17	-0.07	-0.25	0.01
**H3**	0.33	-0.91	-0.02	-0.55
**H9**	-0.33	-0.1	0.13	-0.56
**H11**	-0.31	0.39	0.13	-0.05
**H11a**	-0.4	-0.03	-0.23	-0.19
**H12**	-0.29	-0.02	-0.33	0.01
**H14**	-1.15	-0.69	-0.78	-1.06
**H15**	-0.44	-0.23	-0.29	-0.37
**H18**	0.34	-0.01	-0.15	0.48
**H20**	0.2	-0.29	-0.05	-0.04
**H21**	-0.06	-0.44	-0.4	-0.10

Table S11 ^{1}H chemical shifts of cabotegravir in different solvents as obtained by NMR experiments.

Atom	δ(^{1}H) (DMSO)	δ(^{1}H) (DMSO /D_2O)	δ(^{1}H) (D_2O)	Δδ(^{1}H) (DMSO/D_2O vs. DMSO)	Δδ(^{1}H) (D_2O vs. DMSO)	Δδ(^{1}H) (D_2O vs. (DMSO/D_2O)
**H2**	4.03 (3.66, 4.4)	3.65 (3.65, -)	4.065 (3.75,4.38)	-	0.035	-
**H3**	4.3	-	-	-	-	-
**H9**	8.47	8.24	8.2	-0.23	-0.27	-0.04
**H11**	4.45 (4.01, 4.89)	3.95 (3.95, -)	4.04 (4.04, -)	-	-	-
**H11a**	5.39	5.32	5.41	-0.07	0.02	0.09
**H12**	1.35	1.27	1.33	-0.08	-0.02	0.06
**H14**	10.3	10.38	-	0.08	-	-
**H15**	4.55	4.6	4.56	0.05	0.01	-0.04
**H18**	7.07	6.93	6.92	-0.14	-0.15	-0.01
**H20**	7.24	7.01	-	-0.23	-	-
**H21**	7.4	7.34	7.33	-0.06	-0.07	-0.01

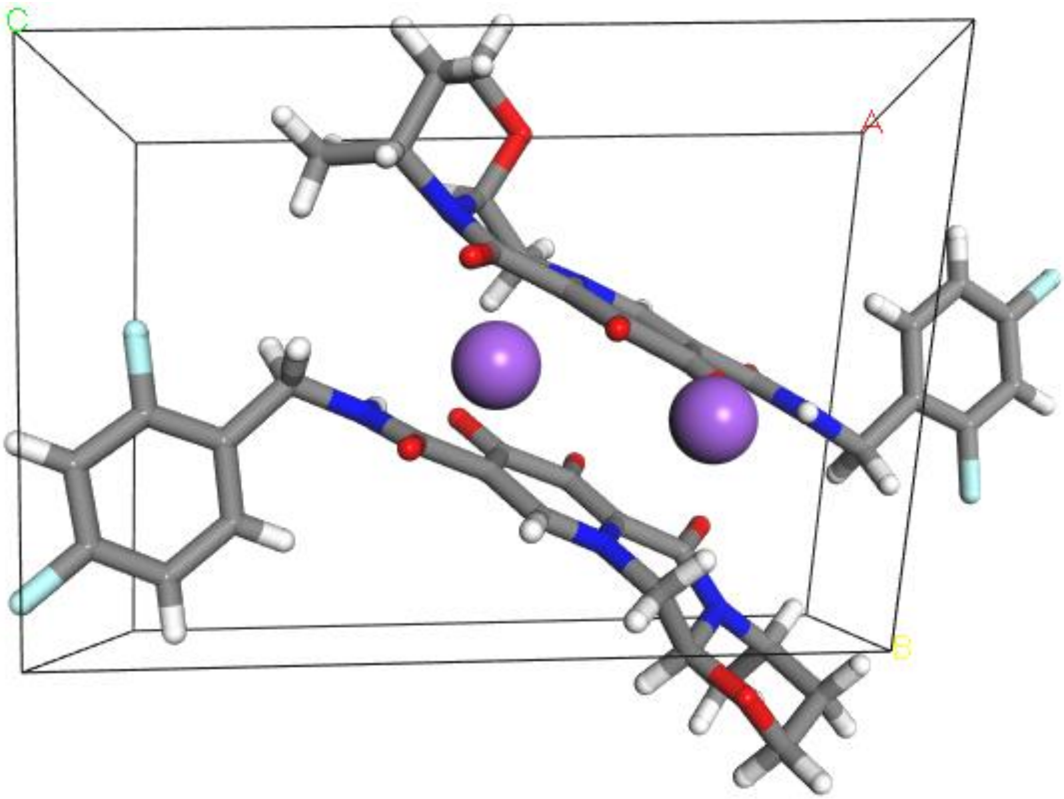

Figure S1. Crystal structure of sodium salt of dolutegravir (CSD Entry: KILNEA).

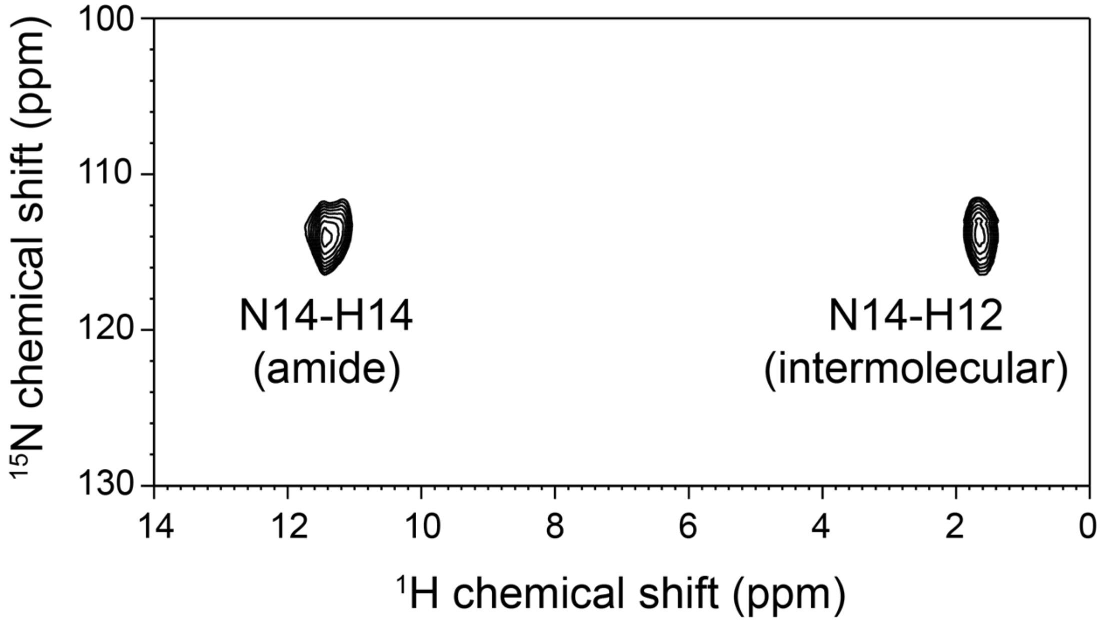

Figure S2. ^{1}H-^{15}N HSQC SSNMR spectra of cabotegravir microcrystalline powder acquired under VFMAS.

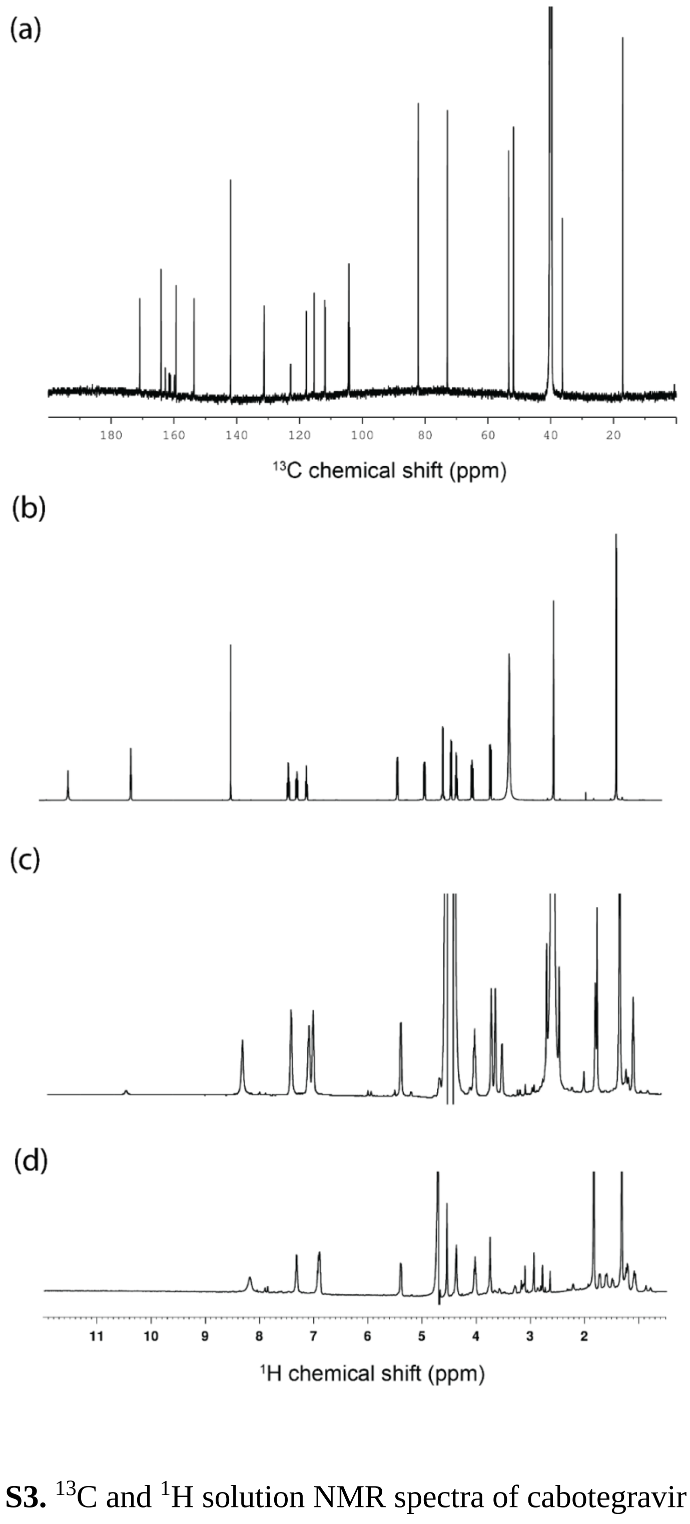

Figure S3. ^{13}C and ^{1}H solution NMR spectra of cabotegravir in different solvents: (a) DMSO solution, (b) DMSO solution, (c) DMSO/D_2O solution, (d) aqueous solution.

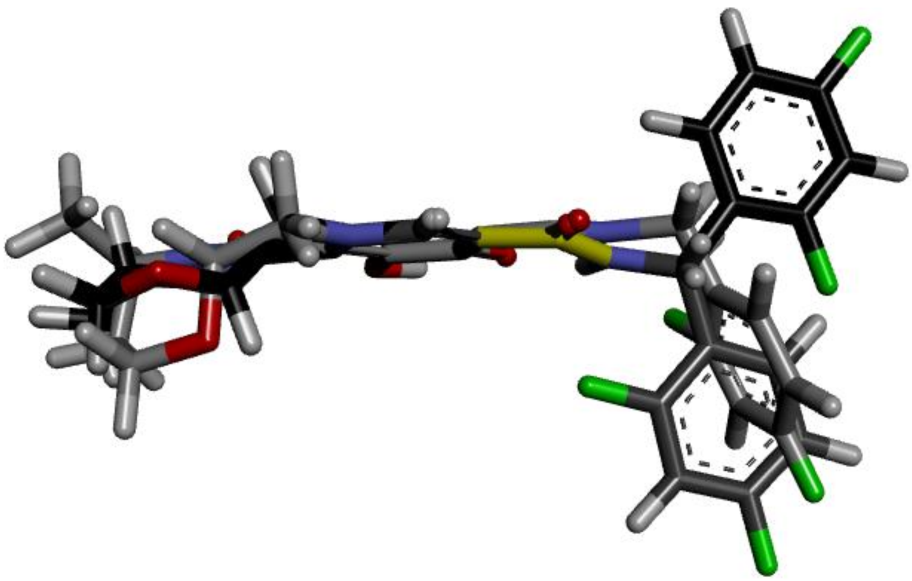

Figure S4. The comparison of the crystal structure of cabotegravir (dark gray), the best computed cabotegravir structural model in aqueous solution (middle gray) and the crystal structure of dolutegravir (light gray).

570.CIF
data_570

_audit_creation_method 'SHELXL-2018/3'
_shelx_SHELXL_version_number '2018/3'
_chemical_name_systematic ?
_chemical_name_common ?
_chemical_melting_point ?
_chemical_formula_moiety 'C19 H17 F2 N3 O5'
_chemical_formula_sum
'C19 H17 F2 N3 O5'
_chemical_formula_weight 405.35

loop_
_atom_type_symbol
_atom_type_description
_atom_type_scat_dispersion_real
_atom_type_scat_dispersion_imag
_atom_type_scat_source
'C' 'C' 0.0181 0.0091
'International Tables Vol C Tables 4.2.6.8 and 6.1.1.4'
'H' 'H' 0.0000 0.0000
'International Tables Vol C Tables 4.2.6.8 and 6.1.1.4'
'N' 'N' 0.0311 0.0180
'International Tables Vol C Tables 4.2.6.8 and 6.1.1.4'

```
 'O'  'O'   0.0492   0.0322
 'International Tables Vol C Tables 4.2.6.8 and 6.1.1.4'
 'F'  'F'   0.0727   0.0534
 'International Tables Vol C Tables 4.2.6.8 and 6.1.1.4'

_space_group_crystal_system       orthorhombic
_space_group_IT_number            19
_space_group_name_H-M_alt         'P 21 21 21'
_space_group_name_Hall            'P 2ac 2ab'

_shelx_space_group_comment
;
The symmetry employed for this shelxl refinement is uniquely defined
by the following loop, which should always be used as a source of
symmetry information in preference to the above space-group names.
They are only intended as comments.
;

loop_
 _space_group_symop_operation_xyz
 'x, y, z'
 '-x+1/2, -y, z+1/2'
 '-x, y+1/2, -z+1/2'
 'x+1/2, -y+1/2, -z'

_cell_length_a                    7.3002(5)
_cell_length_b                    7.30020(10)
_cell_length_c                    32.343(2)
_cell_angle_alpha                 90
_cell_angle_beta                  90
_cell_angle_gamma                 90
_cell_volume                      1723.63(16)
_cell_formula_units_Z             4
_cell_measurement_temperature     182(2)
_cell_measurement_reflns_used     9912
_cell_measurement_theta_min       5.47
_cell_measurement_theta_max       68.82

_exptl_crystal_description        prismatic
_exptl_crystal_colour             colorless
_exptl_crystal_density_meas       ?
_exptl_crystal_density_method     ?
_exptl_crystal_density_diffrn     1.562
_exptl_crystal_F_000              840
_exptl_transmission_factor_min    ?
_exptl_transmission_factor_max    ?
_exptl_crystal_size_max           0.260
_exptl_crystal_size_mid           0.140
_exptl_crystal_size_min           0.080
_exptl_absorpt_coefficient_mu     1.103
_shelx_estimated_absorpt_T_min    0.762
_shelx_estimated_absorpt_T_max    0.917
_exptl_absorpt_correction_type    multi-scan
_exptl_absorpt_correction_T_min   0.6369
```

```
_exptl_absorpt_correction_T_max   0.7531
_exptl_absorpt_process_details    SADABS
_exptl_absorpt_special_details    ?
_diffrn_ambient_temperature       182(2)
_diffrn_radiation_wavelength      1.54178
_diffrn_radiation_type            CuK\a
_diffrn_source                    ?
_diffrn_measurement_device_type   'Bruker APEX-II CCD'
_diffrn_measurement_method        '\f and \w scans'
_diffrn_detector_area_resol_mean  ?
_diffrn_reflns_number             17089
_diffrn_reflns_av_unetI/netI      0.0223
_diffrn_reflns_av_R_equivalents   0.0349
_diffrn_reflns_limit_h_min        -8
_diffrn_reflns_limit_h_max        8
_diffrn_reflns_limit_k_min        -8
_diffrn_reflns_limit_k_max        8
_diffrn_reflns_limit_l_min        -31
_diffrn_reflns_limit_l_max        39
_diffrn_reflns_theta_min          5.471
_diffrn_reflns_theta_max          68.809
_diffrn_reflns_theta_full         67.679
_diffrn_measured_fraction_theta_max   0.993
_diffrn_measured_fraction_theta_full  0.994
_diffrn_reflns_Laue_measured_fraction_max    0.993
_diffrn_reflns_Laue_measured_fraction_full   0.994
_diffrn_reflns_point_group_measured_fraction_max   0.994
_diffrn_reflns_point_group_measured_fraction_full  0.996
_reflns_number_total              3165
_reflns_number_gt                 3114
_reflns_threshold_expression      'I > 2\s(I)'
_reflns_Friedel_coverage          0.698
_reflns_Friedel_fraction_max      0.995
_reflns_Friedel_fraction_full     0.999

_reflns_special_details
;
 Reflections were merged by SHELXL according to the crystal
 class for the calculation of statistics and refinement.

 _reflns_Friedel_fraction is defined as the number of unique
 Friedel pairs measured divided by the number that would be
 possible theoretically, ignoring centric projections and
 systematic absences.
;

_computing_data_collection        'Bruker APEX2'
_computing_cell_refinement        'Bruker SAINT'
_computing_data_reduction         'Bruker SAINT'
_computing_structure_solution     'SHELXS-97 (Sheldrick 2008)'
_computing_structure_refinement   'SHELXL-2018/3 (Sheldrick, 2018)'
_computing_molecular_graphics     'Bruker SHELXTL'
_computing_publication_material   'Bruker SHELXTL'
_refine_special_details           ?
```

_refine_ls_structure_factor_coef Fsqd
_refine_ls_matrix_type   full
_refine_ls_weighting_scheme   calc
_refine_ls_weighting_details
'w=1/[\s^2^(Fo^2^)+(0.0403P)^2^+0.3502P] where P=(Fo^2^+2Fc^2^)/3'
_atom_sites_solution_primary   ?
_atom_sites_solution_secondary   ?
_atom_sites_solution_hydrogens   geom
_refine_ls_hydrogen_treatment   constr
_refine_ls_extinction_method   none
_refine_ls_extinction_coef   .
_refine_ls_abs_structure_details
;
 Flack x determined using 1272 quotients [(I+)-(I-)]/[(I+)+(I-)]
 (Parsons, Flack and Wagner, Acta Cryst. B69 (2013) 249-259).
;
_refine_ls_abs_structure_Flack   0.01(4)
_chemical_absolute_configuration ad
_refine_ls_number_reflns   3165
_refine_ls_number_parameters   262
_refine_ls_number_restraints   0
_refine_ls_R_factor_all   0.0279
_refine_ls_R_factor_gt   0.0274
_refine_ls_wR_factor_ref   0.0730
_refine_ls_wR_factor_gt   0.0722
_refine_ls_goodness_of_fit_ref   1.059
_refine_ls_restrained_S_all   1.059
_refine_ls_shift/su_max   0.001
_refine_ls_shift/su_mean   0.000

loop_
 _atom_site_label
 _atom_site_type_symbol
 _atom_site_fract_x
 _atom_site_fract_y
 _atom_site_fract_z
 _atom_site_U_iso_or_equiv
 _atom_site_adp_type
 _atom_site_occupancy
 _atom_site_site_symmetry_order
 _atom_site_calc_flag
 _atom_site_refinement_flags_posn
 _atom_site_refinement_flags_adp
 _atom_site_refinement_flags_occupancy
 _atom_site_disorder_assembly
 _atom_site_disorder_group
F1 F 0.0164(2) -0.03917(17) 0.51190(4) 0.0444(4) Uani 1 1 d . . . . .
F2 F -0.0042(3) 0.5755(2) 0.55101(4) 0.0567(4) Uani 1 1 d . . . . .
O1 O -0.1161(2) 0.1631(2) 0.35270(4) 0.0335(3) Uani 1 1 d . . . . .
O2 O 0.46392(19) 0.1466(2) 0.35597(4) 0.0268(3) Uani 1 1 d . . . . .
O3 O 0.65882(17) 0.1690(2) 0.28808(4) 0.0293(3) Uani 1 1 d . . . . .
H3A H 0.688729 0.171634 0.313157 0.044 Uiso 1 1 calc R U . . .
O4 O 0.65367(18) 0.2725(2) 0.20616(4) 0.0274(3) Uani 1 1 d . . . . .
O5 O 0.14108(19) 0.1559(2) 0.13474(4) 0.0308(3) Uani 1 1 d . . . . .

N1 N 0.1344(2) 0.0901(2) 0.39038(5) 0.0262(4) Uani 1 1 d . . . . .
H1A H 0.254861 0.086895 0.391341 0.031 Uiso 1 1 calc R U . . .
N2 N 0.1995(2) 0.2090(2) 0.24623(4) 0.0218(3) Uani 1 1 d . . . . .
N3 N 0.4014(2) 0.1581(2) 0.17380(5) 0.0228(3) Uani 1 1 d . . . . .
C1 C 0.0241(3) 0.1844(3) 0.45958(6) 0.0246(4) Uani 1 1 d . . . . .
C2 C 0.0164(3) 0.1402(3) 0.50118(6) 0.0279(4) Uani 1 1 d . . . . .
C3 C 0.0088(3) 0.2672(3) 0.53256(6) 0.0341(5) Uani 1 1 d . . . . .
H3B H 0.004873 0.231832 0.560811 0.041 Uiso 1 1 calc R U . . .
C4 C 0.0071(3) 0.4470(3) 0.52081(6) 0.0339(5) Uani 1 1 d . . . . .
C5 C 0.0151(3) 0.5022(3) 0.48018(7) 0.0337(5) Uani 1 1 d . . . . .
H5A H 0.014757 0.628551 0.473144 0.040 Uiso 1 1 calc R U . . .
C6 C 0.0238(3) 0.3691(3) 0.44990(6) 0.0284(4) Uani 1 1 d . . . . .
H6A H 0.029683 0.405185 0.421715 0.034 Uiso 1 1 calc R U . . .
C7 C 0.0306(3) 0.0366(3) 0.42684(6) 0.0295(4) Uani 1 1 d . . . . .
H7A H 0.086339 -0.075021 0.438853 0.035 Uiso 1 1 calc R U . . .
H7B H -0.096104 0.005798 0.418428 0.035 Uiso 1 1 calc R U . . .
C8 C 0.0523(3) 0.1441(3) 0.35538(5) 0.0231(4) Uani 1 1 d . . . . .
C9 C 0.1775(2) 0.1742(2) 0.31945(5) 0.0218(4) Uani 1 1 d . . . . .
C10 C 0.3732(3) 0.1673(2) 0.32316(5) 0.0218(4) Uani 1 1 d . . . . .
C11 C 0.4756(3) 0.1839(2) 0.28477(6) 0.0221(4) Uani 1 1 d . . . . .
C12 C 0.3908(2) 0.2040(3) 0.24743(6) 0.0210(4) Uani 1 1 d . . . . .
C13 C 0.4951(3) 0.2162(2) 0.20730(6) 0.0222(4) Uani 1 1 d . . . . .
C14 C 0.4603(3) 0.1931(3) 0.13069(5) 0.0241(4) Uani 1 1 d . . . . .
H14A H 0.468605 0.328174 0.125884 0.029 Uiso 1 1 calc R U . . .
C15 C 0.2939(3) 0.1167(3) 0.10801(6) 0.0309(5) Uani 1 1 d . . . . .
H15A H 0.278383 0.177317 0.080857 0.037 Uiso 1 1 calc R U . . .
H15B H 0.306680 -0.016874 0.103606 0.037 Uiso 1 1 calc R U . . .
C16 C 0.2039(3) 0.1151(3) 0.17488(5) 0.0234(4) Uani 1 1 d . . . . .
H16A H 0.183536 -0.017098 0.181576 0.028 Uiso 1 1 calc R U . . .
C17 C 0.1089(3) 0.2367(3) 0.20593(6) 0.0245(4) Uani 1 1 d . . . . .
H17A H 0.118517 0.366621 0.197408 0.029 Uiso 1 1 calc R U . . .
H17B H -0.022478 0.204078 0.207874 0.029 Uiso 1 1 calc R U . . .
C18 C 0.1001(3) 0.1959(3) 0.28102(6) 0.0225(4) Uani 1 1 d . . . . .
H18A H -0.029564 0.201907 0.278994 0.027 Uiso 1 1 calc R U . . .
C19 C 0.6398(3) 0.1031(3) 0.11849(6) 0.0306(5) Uani 1 1 d . . . . .
H19A H 0.668298 0.133257 0.089668 0.046 Uiso 1 1 calc R U . . .
H19B H 0.738163 0.148102 0.136449 0.046 Uiso 1 1 calc R U . . .
H19C H 0.628918 -0.029997 0.121472 0.046 Uiso 1 1 calc R U . . .

loop_
 _atom_site_aniso_label
 _atom_site_aniso_U_11
 _atom_site_aniso_U_22
 _atom_site_aniso_U_33
 _atom_site_aniso_U_23
 _atom_site_aniso_U_13
 _atom_site_aniso_U_12
F1 0.0771(10) 0.0295(6) 0.0266(6) 0.0082(5) 0.0084(6) 0.0046(7)
F2 0.0983(13) 0.0410(7) 0.0310(7) -0.0134(6) -0.0002(8) 0.0023(9)
O1 0.0228(7) 0.0565(10) 0.0212(7) 0.0020(7) 0.0018(5) 0.0003(7)
O2 0.0265(7) 0.0352(7) 0.0187(6) 0.0004(6) -0.0025(5) 0.0018(6)
O3 0.0203(7) 0.0484(9) 0.0193(6) 0.0017(6) -0.0020(5) 0.0015(6)
O4 0.0235(7) 0.0353(8) 0.0235(6) 0.0011(6) 0.0016(5) -0.0056(6)
O5 0.0283(7) 0.0479(9) 0.0161(6) -0.0028(6) -0.0019(5) 0.0008(7)

N1 0.0276(8) 0.0338(9) 0.0173(7) 0.0015(6) 0.0010(6) 0.0018(7)
N2 0.0235(8) 0.0255(8) 0.0164(7) 0.0005(6) -0.0003(6) 0.0010(6)
N3 0.0227(8) 0.0283(8) 0.0174(7) -0.0003(7) 0.0017(6) -0.0013(6)
C1 0.0225(9) 0.0313(10) 0.0199(9) 0.0024(8) 0.0021(7) -0.0004(8)
C2 0.0322(10) 0.0282(10) 0.0234(9) 0.0046(8) 0.0031(8) 0.0019(9)
C3 0.0451(12) 0.0398(12) 0.0174(9) 0.0031(8) 0.0015(9) 0.0043(10)
C4 0.0437(12) 0.0348(11) 0.0232(9) -0.0066(8) 0.0004(9) 0.0015(10)
C5 0.0433(12) 0.0276(10) 0.0304(11) 0.0022(8) 0.0013(10) 0.0009(9)
C6 0.0341(10) 0.0312(10) 0.0199(9) 0.0052(8) 0.0012(8) 0.0007(9)
C7 0.0386(11) 0.0306(10) 0.0192(9) 0.0029(8) 0.0042(9) -0.0032(9)
C8 0.0262(10) 0.0258(9) 0.0174(8) -0.0038(7) 0.0007(7) -0.0005(7)
C9 0.0241(9) 0.0221(9) 0.0192(9) -0.0016(7) 0.0004(7) 0.0002(7)
C10 0.0260(9) 0.0207(9) 0.0187(8) -0.0017(7) -0.0017(7) 0.0019(8)
C11 0.0223(9) 0.0219(8) 0.0219(9) -0.0019(7) 0.0000(7) 0.0006(8)
C12 0.0210(8) 0.0214(9) 0.0205(9) 0.0005(7) 0.0017(7) 0.0004(7)
C13 0.0264(9) 0.0203(8) 0.0199(8) 0.0009(7) 0.0000(7) 0.0009(8)
C14 0.0304(10) 0.0258(9) 0.0162(8) 0.0014(7) 0.0028(7) -0.0008(8)
C15 0.0319(11) 0.0419(12) 0.0190(9) -0.0033(8) 0.0010(8) -0.0025(9)
C16 0.0239(9) 0.0278(9) 0.0184(8) 0.0001(7) -0.0009(7) -0.0022(7)
C17 0.0248(9) 0.0305(10) 0.0182(9) 0.0019(7) -0.0019(7) 0.0027(8)
C18 0.0218(9) 0.0249(9) 0.0208(9) 0.0000(8) 0.0025(7) 0.0016(7)
C19 0.0342(11) 0.0369(11) 0.0206(9) 0.0000(8) 0.0056(8) 0.0024(9)

_geom_special_details
;
 All esds (except the esd in the dihedral angle between two l.s. planes)
 are estimated using the full covariance matrix. The cell esds are taken
 into account individually in the estimation of esds in distances, angles
 and torsion angles; correlations between esds in cell parameters are only
 used when they are defined by crystal symmetry. An approximate (isotropic)
 treatment of cell esds is used for estimating esds involving l.s. planes.
;

loop_
 _geom_bond_atom_site_label_1
 _geom_bond_atom_site_label_2
 _geom_bond_distance
 _geom_bond_site_symmetry_2
 _geom_bond_publ_flag
F1 C2 1.355(2) . ?
F2 C4 1.357(2) . ?
O1 C8 1.240(2) . ?
O2 C10 1.260(2) . ?
O3 C11 1.346(2) . ?
O3 H3A 0.8400 . ?
O4 C13 1.229(2) . ?
O5 C16 1.409(2) . ?
O5 C15 1.440(2) . ?
N1 C8 1.340(2) . ?
N1 C7 1.455(2) . ?
N1 H1A 0.8800 . ?
N2 C18 1.342(2) . ?
N2 C12 1.397(2) . ?
N2 C17 1.476(2) . ?

N3 C13 1.350(2) . ?
N3 C16 1.476(2) . ?
N3 C14 1.481(2) . ?
C1 C6 1.384(3) . ?
C1 C2 1.385(3) . ?
C1 C7 1.513(3) . ?
C2 C3 1.375(3) . ?
C3 C4 1.367(3) . ?
C3 H3B 0.9500 . ?
C4 C5 1.376(3) . ?
C5 C6 1.381(3) . ?
C5 H5A 0.9500 . ?
C6 H6A 0.9500 . ?
C7 H7A 0.9900 . ?
C7 H7B 0.9900 . ?
C8 C9 1.495(2) . ?
C9 C18 1.374(2) . ?
C9 C10 1.435(3) . ?
C10 C11 1.454(3) . ?
C11 C12 1.365(3) . ?
C12 C13 1.507(3) . ?
C14 C19 1.518(3) . ?
C14 C15 1.525(3) . ?
C14 H14A 1.0000 . ?
C15 H15A 0.9900 . ?
C15 H15B 0.9900 . ?
C16 C17 1.509(3) . ?
C16 H16A 1.0000 . ?
C17 H17A 0.9900 . ?
C17 H17B 0.9900 . ?
C18 H18A 0.9500 . ?
C19 H19A 0.9800 . ?
C19 H19B 0.9800 . ?
C19 H19C 0.9800 . ?

loop_
 _geom_angle_atom_site_label_1
 _geom_angle_atom_site_label_2
 _geom_angle_atom_site_label_3
 _geom_angle
 _geom_angle_site_symmetry_1
 _geom_angle_site_symmetry_3
 _geom_angle_publ_flag
C11 O3 H3A 109.5 . . ?
C16 O5 C15 105.01(14) . . ?
C8 N1 C7 122.03(17) . . ?
C8 N1 H1A 119.0 . . ?
C7 N1 H1A 119.0 . . ?
C18 N2 C12 121.00(15) . . ?
C18 N2 C17 120.50(15) . . ?
C12 N2 C17 118.45(15) . . ?
C13 N3 C16 122.86(15) . . ?
C13 N3 C14 123.66(15) . . ?
C16 N3 C14 110.05(14) . . ?

C6 C1 C2 116.53(18) . . ?
C6 C1 C7 122.45(17) . . ?
C2 C1 C7 121.02(18) . . ?
F1 C2 C3 117.55(17) . . ?
F1 C2 C1 118.28(17) . . ?
C3 C2 C1 124.17(19) . . ?
C4 C3 C2 116.26(18) . . ?
C4 C3 H3B 121.9 . . ?
C2 C3 H3B 121.9 . . ?
F2 C4 C3 117.69(19) . . ?
F2 C4 C5 119.2(2) . . ?
C3 C4 C5 123.14(19) . . ?
C4 C5 C6 118.23(19) . . ?
C4 C5 H5A 120.9 . . ?
C6 C5 H5A 120.9 . . ?
C5 C6 C1 121.68(18) . . ?
C5 C6 H6A 119.2 . . ?
C1 C6 H6A 119.2 . . ?
N1 C7 C1 113.07(16) . . ?
N1 C7 H7A 109.0 . . ?
C1 C7 H7A 109.0 . . ?
N1 C7 H7B 109.0 . . ?
C1 C7 H7B 109.0 . . ?
H7A C7 H7B 107.8 . . ?
O1 C8 N1 122.36(17) . . ?
O1 C8 C9 122.38(16) . . ?
N1 C8 C9 115.22(16) . . ?
C18 C9 C10 119.29(16) . . ?
C18 C9 C8 117.93(16) . . ?
C10 C9 C8 122.61(16) . . ?
O2 C10 C9 126.72(17) . . ?
O2 C10 C11 117.33(16) . . ?
C9 C10 C11 115.94(16) . . ?
O3 C11 C12 121.99(16) . . ?
O3 C11 C10 115.84(15) . . ?
C12 C11 C10 122.09(17) . . ?
C11 C12 N2 118.73(16) . . ?
C11 C12 C13 122.64(17) . . ?
N2 C12 C13 118.62(16) . . ?
O4 C13 N3 123.93(17) . . ?
O4 C13 C12 121.41(16) . . ?
N3 C13 C12 114.65(16) . . ?
N3 C14 C19 114.89(15) . . ?
N3 C14 C15 99.11(14) . . ?
C19 C14 C15 113.85(16) . . ?
N3 C14 H14A 109.5 . . ?
C19 C14 H14A 109.5 . . ?
C15 C14 H14A 109.5 . . ?
O5 C15 C14 104.82(14) . . ?
O5 C15 H15A 110.8 . . ?
C14 C15 H15A 110.8 . . ?
O5 C15 H15B 110.8 . . ?
C14 C15 H15B 110.8 . . ?
H15A C15 H15B 108.9 . . ?

O5 C16 N3 104.53(14) . . ?
O5 C16 C17 109.83(15) . . ?
N3 C16 C17 109.84(15) . . ?
O5 C16 H16A 110.8 . . ?
N3 C16 H16A 110.8 . . ?
C17 C16 H16A 110.8 . . ?
N2 C17 C16 107.51(15) . . ?
N2 C17 H17A 110.2 . . ?
C16 C17 H17A 110.2 . . ?
N2 C17 H17B 110.2 . . ?
C16 C17 H17B 110.2 . . ?
H17A C17 H17B 108.5 . . ?
N2 C18 C9 122.95(17) . . ?
N2 C18 H18A 118.5 . . ?
C9 C18 H18A 118.5 . . ?
C14 C19 H19A 109.5 . . ?
C14 C19 H19B 109.5 . . ?
H19A C19 H19B 109.5 . . ?
C14 C19 H19C 109.5 . . ?
H19A C19 H19C 109.5 . . ?
H19B C19 H19C 109.5 . . ?

loop_
_geom_torsion_atom_site_label_1
_geom_torsion_atom_site_label_2
_geom_torsion_atom_site_label_3
_geom_torsion_atom_site_label_4
_geom_torsion
_geom_torsion_site_symmetry_1
_geom_torsion_site_symmetry_2
_geom_torsion_site_symmetry_3
_geom_torsion_site_symmetry_4
_geom_torsion_publ_flag
C6 C1 C2 F1 180.00(19) . . . . ?
C7 C1 C2 F1 0.6(3) . . . . ?
C6 C1 C2 C3 -0.1(3) . . . . ?
C7 C1 C2 C3 -179.5(2) . . . . ?
F1 C2 C3 C4 -179.4(2) . . . . ?
C1 C2 C3 C4 0.7(4) . . . . ?
C2 C3 C4 F2 178.7(2) . . . . ?
C2 C3 C4 C5 -0.9(4) . . . . ?
F2 C4 C5 C6 -179.0(2) . . . . ?
C3 C4 C5 C6 0.5(4) . . . . ?
C4 C5 C6 C1 0.1(4) . . . . ?
C2 C1 C6 C5 -0.3(3) . . . . ?
C7 C1 C6 C5 179.0(2) . . . . ?
C8 N1 C7 C1 -101.8(2) . . . . ?
C6 C1 C7 N1 32.6(3) . . . . ?
C2 C1 C7 N1 -148.08(19) . . . . ?
C7 N1 C8 O1 4.7(3) . . . . ?
C7 N1 C8 C9 -173.43(16) . . . . ?
O1 C8 C9 C18 -10.1(3) . . . . ?
N1 C8 C9 C18 168.05(17) . . . . ?
O1 C8 C9 C10 174.70(19) . . . . ?

N1 C8 C9 C10 -7.2(3) . . . . ?
C18 C9 C10 O2 -179.37(18) . . . . ?
C8 C9 C10 O2 -4.2(3) . . . . ?
C18 C9 C10 C11 -0.2(3) . . . . ?
C8 C9 C10 C11 174.96(16) . . . . ?
O2 C10 C11 O3 2.2(2) . . . . ?
C9 C10 C11 O3 -177.07(16) . . . . ?
O2 C10 C11 C12 179.00(18) . . . . ?
C9 C10 C11 C12 -0.3(3) . . . . ?
O3 C11 C12 N2 176.70(17) . . . . ?
C10 C11 C12 N2 0.1(3) . . . . ?
O3 C11 C12 C13 -1.7(3) . . . . ?
C10 C11 C12 C13 -178.30(16) . . . . ?
C18 N2 C12 C11 0.6(3) . . . . ?
C17 N2 C12 C11 178.06(16) . . . . ?
C18 N2 C12 C13 179.01(16) . . . . ?
C17 N2 C12 C13 -3.5(2) . . . . ?
C16 N3 C13 O4 -171.40(17) . . . . ?
C14 N3 C13 O4 -14.4(3) . . . . ?
C16 N3 C13 C12 9.7(3) . . . . ?
C14 N3 C13 C12 166.71(16) . . . . ?
C11 C12 C13 O4 -26.0(3) . . . . ?
N2 C12 C13 O4 155.58(18) . . . . ?
C11 C12 C13 N3 152.89(18) . . . . ?
N2 C12 C13 N3 -25.5(2) . . . . ?
C13 N3 C14 C19 64.4(2) . . . . ?
C16 N3 C14 C19 -136.01(17) . . . . ?
C13 N3 C14 C15 -173.83(18) . . . . ?
C16 N3 C14 C15 -14.28(19) . . . . ?
C16 O5 C15 C14 -42.22(19) . . . . ?
N3 C14 C15 O5 33.47(18) . . . . ?
C19 C14 C15 O5 155.95(16) . . . . ?
C15 O5 C16 N3 31.90(19) . . . . ?
C15 O5 C16 C17 149.68(16) . . . . ?
C13 N3 C16 O5 149.70(17) . . . . ?
C14 N3 C16 O5 -10.0(2) . . . . ?
C13 N3 C16 C17 31.9(2) . . . . ?
C14 N3 C16 C17 -127.83(16) . . . . ?
C18 N2 C17 C16 -138.09(17) . . . . ?
C12 N2 C17 C16 44.4(2) . . . . ?
O5 C16 C17 N2 -171.32(14) . . . . ?
N3 C16 C17 N2 -56.88(19) . . . . ?
C12 N2 C18 C9 -1.0(3) . . . . ?
C17 N2 C18 C9 -178.51(17) . . . . ?
C10 C9 C18 N2 0.8(3) . . . . ?
C8 C9 C18 N2 -174.53(17) . . . . ?

loop_
 _geom_hbond_atom_site_label_D
 _geom_hbond_atom_site_label_H
 _geom_hbond_atom_site_label_A
 _geom_hbond_distance_DH
 _geom_hbond_distance_HA
 _geom_hbond_distance_DA

```
 _geom_hbond_angle_DHA
 _geom_hbond_site_symmetry_A
 _geom_hbond_publ_flag
O3 H3A O1  0.84 1.92 2.6587(18) 146.8 1_655 yes
O3 H3A O2  0.84 2.16 2.6215(18) 114.9 . yes
N1 H1A O2  0.88 1.96 2.682(2) 138.8 . yes

_refine_diff_density_max    0.157
_refine_diff_density_min   -0.172
_refine_diff_density_rms    0.036

_shelx_res_file
;
TITL GSK744 in P2(1)2(1)2(1)
    570.res
    created by SHELXL-2018/3 at 13:58:58 on 02-Jan-2019
CELL  1.54178   7.3002   7.3002  32.3426   90.000   90.000   90.000
ZERR    4.000   0.0005   0.0001   0.0021    0.000    0.000    0.000
LATT -1
SYMM 1/2-X, -Y, 1/2+Z
SYMM -X, 1/2+Y, 1/2-Z
SYMM 1/2+X, 1/2-Y, -Z
SFAC C H N O F
UNIT 76 68 12 20 8
TEMP -90.730
L.S. 4

ACTA
BOND $H
CONF
EQIV $1 x+1, y, z
HTAB O3 O1_$1
HTAB O3 O2
HTAB N1 O2
SIZE 0.08 0.14 0.26
FMAP 2
PLAN 10
WGHT    0.040300    0.350200
FVAR       0.93199
F1    5    0.016443   -0.039173    0.511896    11.00000    0.07714    0.02946 =
         0.02657    0.00817    0.00840    0.00455
F2    5   -0.004212    0.575509    0.551009    11.00000    0.09829    0.04096 =
         0.03100   -0.01336   -0.00023    0.00232
O1    4   -0.116125    0.163073    0.352701    11.00000    0.02276    0.05649 =
         0.02118    0.00203    0.00184    0.00035
O2    4    0.463921    0.146615    0.355974    11.00000    0.02647    0.03521 =
         0.01872    0.00042   -0.00251    0.00180
O3    4    0.658824    0.169045    0.288085    11.00000    0.02031    0.04843 =
         0.01931    0.00174   -0.00197    0.00149
AFIX  83
H3A   2    0.688729    0.171634    0.313157    11.00000   -1.50000
AFIX   0
O4    4    0.653674    0.272540    0.206158    11.00000    0.02351    0.03526 =
         0.02345    0.00105    0.00160   -0.00560
```

```
O5    4    0.141079    0.155907    0.134745    11.00000    0.02835    0.04792 =
         0.01615   -0.00279   -0.00195    0.00081
N1    3    0.134435    0.090094    0.390379    11.00000    0.02757    0.03379 =
         0.01725    0.00151    0.00099    0.00183
AFIX  43
H1A   2    0.254861    0.086895    0.391341    11.00000   -1.20000
AFIX   0
N2    3    0.199543    0.209005    0.246231    11.00000    0.02353    0.02547 =
         0.01641    0.00053   -0.00033    0.00103
N3    3    0.401392    0.158117    0.173796    11.00000    0.02267    0.02832 =
         0.01740   -0.00029    0.00172   -0.00133
C1    1    0.024147    0.184408    0.459584    11.00000    0.02247    0.03133 =
         0.01993    0.00240    0.00210   -0.00044
C2    1    0.016379    0.140236    0.501178    11.00000    0.03218    0.02815 =
         0.02337    0.00464    0.00306    0.00193
C3    1    0.008757    0.267174    0.532556    11.00000    0.04514    0.03979 =
         0.01738    0.00314    0.00150    0.00432
AFIX  43
H3B   2    0.004873    0.231832    0.560811    11.00000   -1.20000
AFIX   0
C4    1    0.007085    0.446980    0.520810    11.00000    0.04372    0.03477 =
         0.02320   -0.00656    0.00041    0.00148
C5    1    0.015142    0.502212    0.480184    11.00000    0.04329    0.02758 =
         0.03035    0.00216    0.00125    0.00088
AFIX  43
H5A   2    0.014757    0.628551    0.473144    11.00000   -1.20000
AFIX   0
C6    1    0.023807    0.369077    0.449904    11.00000    0.03410    0.03124 =
         0.01994    0.00516    0.00116    0.00065
AFIX  43
H6A   2    0.029683    0.405185    0.421715    11.00000   -1.20000
AFIX   0
C7    1    0.030610    0.036564    0.426837    11.00000    0.03859    0.03064 =
         0.01922    0.00292    0.00421   -0.00318
AFIX  23
H7A   2    0.086339   -0.075021    0.438853    11.00000   -1.20000
H7B   2   -0.096104    0.005798    0.418428    11.00000   -1.20000
AFIX   0
C8    1    0.052294    0.144122    0.355379    11.00000    0.02618    0.02576 =
         0.01738   -0.00384    0.00075   -0.00048
C9    1    0.177526    0.174164    0.319446    11.00000    0.02414    0.02205 =
         0.01922   -0.00160    0.00038    0.00020
C10   1    0.373247    0.167317    0.323160    11.00000    0.02605    0.02066 =
         0.01874   -0.00172   -0.00171    0.00190
C11   1    0.475635    0.183925    0.284768    11.00000    0.02234    0.02191 =
         0.02195   -0.00186    0.00003    0.00062
C12   1    0.390837    0.204031    0.247433    11.00000    0.02101    0.02140 =
         0.02051    0.00049    0.00171    0.00036
C13   1    0.495054    0.216248    0.207301    11.00000    0.02636    0.02028 =
         0.01994    0.00091   -0.00003    0.00090
C14   1    0.460328    0.193111    0.130693    11.00000    0.03041    0.02576 =
         0.01621    0.00142    0.00280   -0.00078
AFIX  13
H14A  2    0.468605    0.328174    0.125884    11.00000   -1.20000
```

```
AFIX   0
C15   1    0.293948    0.116719    0.108014    11.00000    0.03186    0.04192 =
        0.01898   -0.00331    0.00104   -0.00252
AFIX  23
H15A  2    0.278383    0.177317    0.080857    11.00000   -1.20000
H15B  2    0.306680   -0.016874    0.103606    11.00000   -1.20000
AFIX   0
C16   1    0.203863    0.115080    0.174880    11.00000    0.02392    0.02784 =
        0.01841    0.00008   -0.00092   -0.00222
AFIX  13
H16A  2    0.183536   -0.017098    0.181576    11.00000   -1.20000
AFIX   0
C17   1    0.108864    0.236734    0.205935    11.00000    0.02478    0.03053 =
        0.01817    0.00194   -0.00190    0.00274
AFIX  23
H17A  2    0.118517    0.366621    0.197408    11.00000   -1.20000
H17B  2   -0.022478    0.204078    0.207874    11.00000   -1.20000
AFIX   0
C18   1    0.100119    0.195894    0.281023    11.00000    0.02180    0.02492 =
        0.02078    0.00002    0.00252    0.00155
AFIX  43
H18A  2   -0.029564    0.201907    0.278994    11.00000   -1.20000
AFIX   0
C19   1    0.639831    0.103147    0.118489    11.00000    0.03421    0.03693 =
        0.02062    0.00002    0.00555    0.00236
AFIX  33
H19A  2    0.668298    0.133257    0.089668    11.00000   -1.50000
H19B  2    0.738163    0.148102    0.136449    11.00000   -1.50000
H19C  2    0.628918   -0.029997    0.121472    11.00000   -1.50000

AFIX   0
HKLF 4




REM  GSK744 in P2(1)2(1)2(1)
REM wR2 = 0.0730, GooF = S = 1.059, Restrained GooF = 1.059 for all data
REM R1 = 0.0274 for 3114 Fo > 4sig(Fo) and 0.0279 for all 3165 data
REM 262 parameters refined using 0 restraints

END

WGHT      0.0403      0.3502

REM Highest difference peak  0.157,  deepest hole -0.172,  1-sigma level  0.036
Q1    1   0.4407  0.1689  0.1538  11.00000  0.05    0.16
Q2    1   0.4416  0.2155  0.2299  11.00000  0.05    0.15
Q3    1   0.1538  0.1690  0.1924  11.00000  0.05    0.15
Q4    1   0.6253  0.0833  0.0827  11.00000  0.05    0.14
Q5    1   0.0577  0.1639  0.4779  11.00000  0.05    0.13
Q6    1   0.7250  0.2382  0.1735  11.00000  0.05    0.13
Q7    1   0.2560  0.1247  0.3217  11.00000  0.05    0.13
Q8    1   0.0728  0.4627  0.5002  11.00000  0.05    0.13
```

Q9 1 0.1242 0.1580 0.3344 11.00000 0.05 0.13
Q10 1 0.0809 0.2655 0.4547 11.00000 0.05 0.12
;
_shelx_res_checksum 51281
;
_shelx_hkl_checksum 2642

checkCIF/PLATON report

Structure factors have been supplied for datablock(s) 570

THIS REPORT IS FOR GUIDANCE ONLY. IF USED AS PART OF A REVIEW PROCEDURE FOR PUBLICATION, IT SHOULD NOT REPLACE THE EXPERTISE OF AN EXPERIENCED CRYSTALLOGRAPHIC REFEREE.

No syntax errors found. CIF dictionary Interpreting this report

Datablock: 570

Bond precision: C-C = 0.0029 A Wavelength=1.54178

Cell:	a=7.3002(5)	b=7.3002(1)	c=32.343(2)
	alpha=90	beta=90	gamma=90
Temperature:	182 K		

	Calculated	Reported
Volume	1723.65(16)	1723.63(16)
Space group	P 21 21 21	P 21 21 21
Hall group	P 2ac 2ab	P 2ac 2ab
Moiety formula	C19 H17 F2 N3 O5	C19 H17 F2 N3 O5
Sum formula	C19 H17 F2 N3 O5	C19 H17 F2 N3 O5
Mr	405.36	405.35
Dx,g cm-3	1.562	1.562
Z	4	4
Mu (mm-1)	1.103	1.103
F000	840.0	840.0
F000'	843.17	
h,k,lmax	8,8,39	8,8,39
Nref	3185[1877]	3165
Tmin,Tmax	0.831,0.916	0.637,0.753
Tmin'	0.751	

Correction method= # Reported T Limits: Tmin=0.637 Tmax=0.753
AbsCorr = MULTI-SCAN

Data completeness= 1.69/0.99 Theta(max)= 68.809

R(reflections)= 0.0274(3114) wR2(reflections)= 0.0730(3165)

S = 1.059 Npar= 262

The following ALERTS were generated. Each ALERT has the format
test-name_ALERT_alert-type_alert-level.
Click on the hyperlinks for more details of the test.

Alert level B

SYMMS02_ALERT_1_B The unit-cell lengths a and b should not be equal for anorthorhombic cell

```
Cell       7.3002    7.3002   32.3430
Angles    90.0000   90.0000   90.0000
```

Alert level C

PLAT089_ALERT_3_C Poor Data / Parameter Ratio (Zmax < 18) .... 7.11 Note PLAT911_ALERT_3_C Missing FCF Refl Between Thmin & STh/L= 0.600 10 Report
PLAT913_ALERT_3_C Missing # of Very Strong Reflections in FCF .... 6 Note

Alert level G

PLAT007_ALERT_5_G Number of Unrefined Donor-H Atoms .... 2 ReportPLAT398_ALERT_2_G Deviating C-O-C Angle From 120 for O5 . 105.0 Degree PLAT883_ALERT_1_G No Info/Value for _atom_sites_solution_primary . Please Do !
PLAT899_ALERT_4_G SHELXL2018 is Deprecated and Succeeded by SHELXL 2019/3 Note
PLAT910_ALERT_3_G Missing # of FCF Reflection(s) Below Theta(Min). 1 Note
PLAT912_ALERT_4_G Missing # of FCF Reflections Above STh/L= 0.600 1 Note
PLAT955_ALERT_1_G Reported (CIF) and Actual (FCF) Lmax Differ by . 1 Units
PLAT978_ALERT_2_G Number C-C Bonds with Positive Residual Density. 10 Info

0 **ALERT level A** = Most likely a serious problem - resolve or explain
1 **ALERT level B** = A potentially serious problem, consider carefully
3 **ALERT level C** = Check. Ensure it is not caused by an omission or oversight
8 **ALERT level G** = General information/check it is not something unexpected

3 ALERT type 1 CIF construction/syntax error, inconsistent or missing data
2 ALERT type 2 Indicator that the structure model may be wrong or deficient
4 ALERT type 3 Indicator that the structure quality may be low
2 ALERT type 4 Improvement, methodology, query or suggestion
1 ALERT type 5 Informative message, check

PLATON version of 06/07/2023; check.def file version of 30/06/2023

Datablock 570 - ellipsoid plot

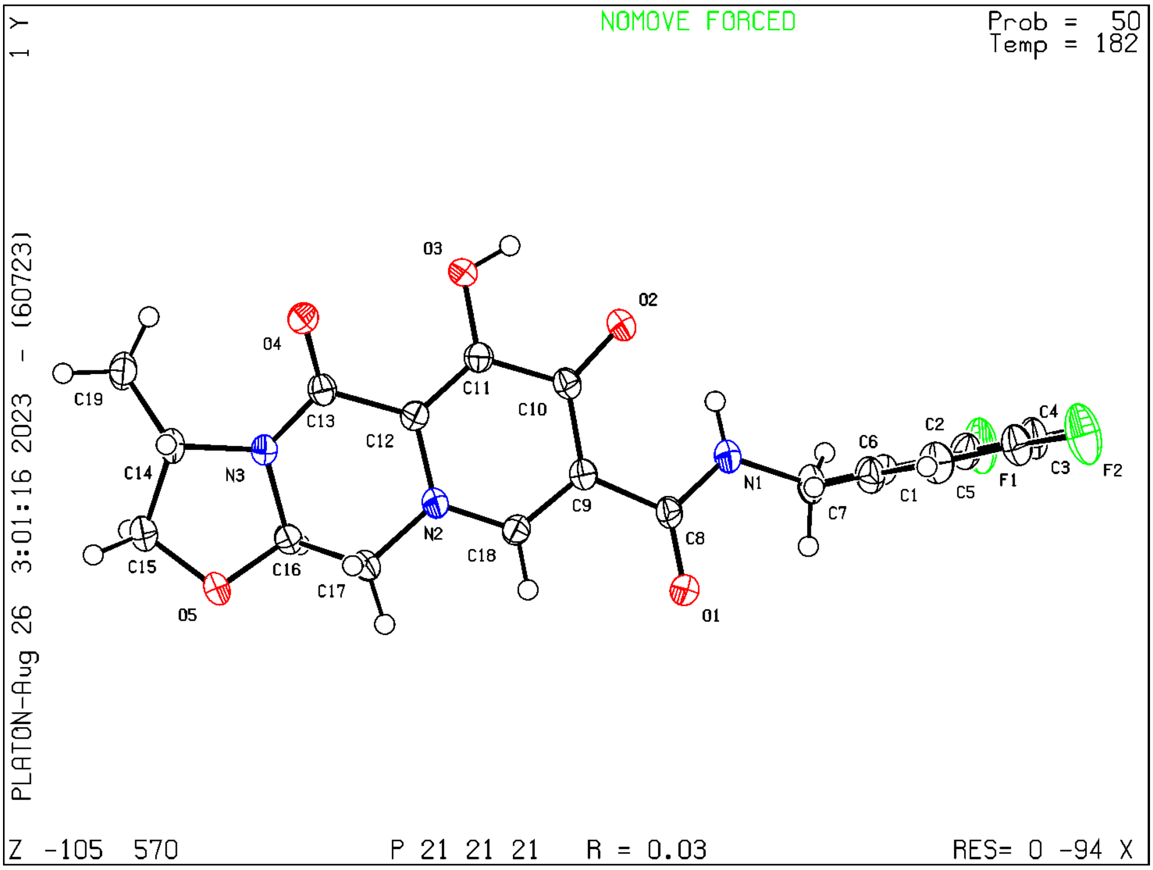

Supplement: Supplementary file 1 [file molecules-29-00376-s001.zip › molecules-2812068-supplementary.pdf]
